# Population genetic structure and demography of *Magnolia kobus*: variety *borealis* is not supported genetically

**DOI:** 10.1007/s10265-019-01134-6

**Published:** 2019-09-05

**Authors:** Ichiro Tamaki, Naomichi Kawashima, Suzuki Setsuko, Jung-Hyun Lee, Akemi Itaya, Kyohei Yukitoshi, Nobuhiro Tomaru

**Affiliations:** 1 Gifu Academy of Forest Science and Culture, 88 Sodai, Mino, Gifu 501-3714 Japan; 2 Graduate School of Bioagricultural Sciences, Nagoya University, Furo-cho, Chikusa-ku, Nagoya, 464-8601 Japan; 3 Department of Forest Molecular Genetics and Biotechnology, Forestry and Forest Products Research Institute, Forest Research and Management Organization, 1 Matsunosato, Tsukuba, Ibaraki 305-8687 Japan; 4 Department of Biology Education, Chonnam National University, 77 Yongbong-ro, Buk-gu, Gwangju, 500-757 Republic of Korea; 5 Graduate School of Bioresources, Mie University, 1577 Kurimamachiya, Tsu, Mie 514-8507 Japan; 6 Present Address: Mie Prefecture Forestry Research Institute, 3769-1 Nihongi, Hakusan-cho, Tsu, Mie 515-2602 Japan

**Keywords:** Approximate Bayesian computation, Chloroplast DNA sequences, Conservation, Ecological niche modeling, Leaf morphology, Microsatellites

## Abstract

Species delimitations by morphological and by genetic markers are not always congruent. *Magnolia kobus* consists of two morphologically different varieties, *kobus* and *borealis*. The latter variety is characterized by larger leaves than the former. For the conservation of *M. kobus* genetic resources in natural forests, the relationships between morphological and genetic variation should be clarified. We investigated variations in nuclear microsatellites, chloroplast DNA (cpDNA) sequences and leaf morphological traits in 23 populations of *M. kobus* over the range of species. Two genetically divergent lineages, northern and southern were detected and their geographical boundary was estimated to be at 39°N. The northern lineage consisted of two genetic clusters and a single cpDNA haplotype, while the southern one had multiple genetic clusters and cpDNA haplotypes. The northern lineage showed significantly lower genetic diversity than the southern. Approximate Bayesian computation indicated that the northern and southern lineages had experienced, respectively, population expansion and long-term stable population size. The divergence time between the two lineages was estimated to be 565,000 years ago and no signature of migration between the two lineages after divergence was detected. Ecological niche modeling showed that the potential distribution area in northern Japan at the last glacial maximum was very small. It is thus considered that the two lineages have experienced different population histories over several glacial-inter-glacial cycles. Individuals of populations in the central to northern part of Honshu on the Sea of Japan side and in Hokkaido had large leaf width and area. These leaf characteristics corresponded with those of variety *borealis*. However, the delimitation of the northern and southern lineages detected by genetic markers (39°N) was not congruent with that detected by leaf morphologies (36°N). It is therefore suggested that variety *borealis* is not supported genetically and the northern and southern lineages should be considered separately when identifying conservation units based not on morphology but on genetic markers.

**Electronic supplementary material:**

The online version of this article (10.1007/s10265-019-01134-6) contains supplementary material, which is available to authorized users.

## Introduction

Tree species that are widely distributed along the Japanese archipelago show significant genetic differentiation for neutral genetic markers between the Sea of Japan and Pacific Ocean sides (*Fagus crenata*, Hiraoka and Tomaru 2009; *Cryptomeria japonica*, Tsumura et al. 2014) and/or between the north and south (*Kalopanax septemlobus*, Sakaguchi et al. 2011; *Quercus aliena*, San Jose-Maldia et al. 2017; *Magnolia salicifolia*, Tamaki et al. 2018). However, the boundaries of genetic differentiation are not always the same among species. This may be mainly due to the differences in locations of refugia during the glacial period among species. Broad-leaved tree species growing along the Sea of Japan side of the Japanese archipelago are often characterized by large-wide-thin leaves, while related species growing in the Pacific Ocean side are characterized by small-narrow-thick leaves (Hotta 1974). The main factor generating these differences is considered to be adaptation to dryness on the Pacific Ocean side during the flushing period (Hotta 1974). Even within a species that is widely distributed along the Japanese archipelago, latitudinal clines of leaf area and leaf width can be detected (Hagiwara 1977; Koyama et al. 2002; Tamaki et al. 2018). Provenance tests on *Fagus crenata* have indicated that variations in leaf morphology and physiology are based not on phenotypic plasticity but on the genetic make-up of individuals (Hashizume et al. 1997; Koike and Maruyama 1998). However, delimitation based on morphological traits and that determined from genetic structure do not always accord (Duminil and Di Michele 2009). It is therefore necessary to clarify the relationships between morphological traits reflecting physiological adaptation to environment, and genetic structure, when considering conservation of genetic resources of forest trees that are broadly distributed along the Japanese archipelago.

*Magnolia kobus* DC., which belongs to the Magnoliaceae, is a major tree species in temperate forests, and it is distributed in Hokkaido, Honshu and Kyushu Islands of Japan and Jeju Island of Korea (Ueda 2006). There are two varieties, *kobus* and *borealis*. Variety *borealis*, which is characterized by larger leaves and flowers than variety *kobus*, is distributed from central to northern Honshu on the Sea of Japan side and on Hokkaido (Ohashi 2015). According to Ohashi (2015), the ranges of leaf length for varieties *kobus* and *borealis* overlap each other (6–15 cm and 10–20 cm, respectively), whereas those of leaf width do not (3–6 cm and 6–10 cm, respectively). Thus, the leaf width could become a key to distinguish the two varieties. However, Callaway (1994) points out that the morphologies of variety *borealis* are not always consistent even within an individual and thus recognizing it as a separate variety does not appear justified. Moreover, the Flora of Japan, which is one of the most authoritative catalogs of Japanese plants, does not treat the variety *borealis* as a distinct variety and treats as one of the synonyms (Ueda 2006), and this may be due to its morphological ambiguity. Recently, Tamaki et al. (2018) have reported that *M. salicifolia*, which is a species related to *M. kobus*, diverges both morphologically and genetically between northern and southern lineages. Accordingly, also in *M. kobus*, the relationships between morphological and genetic variations should be clarified.

*Magnolia kobus* is popular as an ornamental tree due to its beautiful flowers and tolerance of vehicle emissions, and it is planted at roadsides all over Japan. It is also planted when restoring natural deciduous broad-leaved forests (Takasuna and Takayama 2011). The presence within a natural forest of seedlings that have escaped from trees planted near the forest is frequently reported (Fujii 1997; Ishida et al. 2008; Tamaki et al. 2016). Planting without considering the origin of individuals may cause serious genetic disruptions of the genetic resources of *M. kobus* growing in natural forests (Lefèvre 2004; Potts et al. 2003). As the Japanese archipelago is latitudinally long, there is some degree of latitudinal climatic heterogeneity among the habitats of tree species. Moreover, there are climatic differences between the Sea of Japan side and the Pacific Ocean side. Studies of tree species broadly distributed in the Japanese archipelago have reported environmental incongruence among trees that were planted in different sites from those where they originated. Reciprocal transplanting of *Pinus densiflora* lineages between its natural northern and southern habitats revealed that the southern lineage was at a disadvantage in terms of survival and growth when it was transplanted northwards (Nagamitsu et al. 2015). Similarly, a reciprocal transplant of *F. crenata* lineages between the Sea of Japan side and the Pacific Ocean side indicated that both lineages had significant home site advantages with respect to both survival and growth (Koyama 2012). The direction and extent of environmental incongruences brought about by genetic disturbance vary among tree species. It is therefore necessary to determine conservation units carefully, taking into account genetic and ecological information about the target species.

In this study we investigated genetic variation in nuclear microsatellites and chloroplast DNA sequences, and leaf morphological traits, in *M. kobus* populations across the distribution range. We performed approximate Bayesian computation and ecological niche modeling. The specific objectives of this study are (1) to clarify the genetic diversity and structure of *M. kobus* natural populations over the range of the species; (2) to assess the existence of variety *borealis* based on leaf morphological and genetic traits and (3) to infer how best to conserve genetic resources of *M. kobus*.

## Materials and methods

### Sample collection

**Fig. 1:**
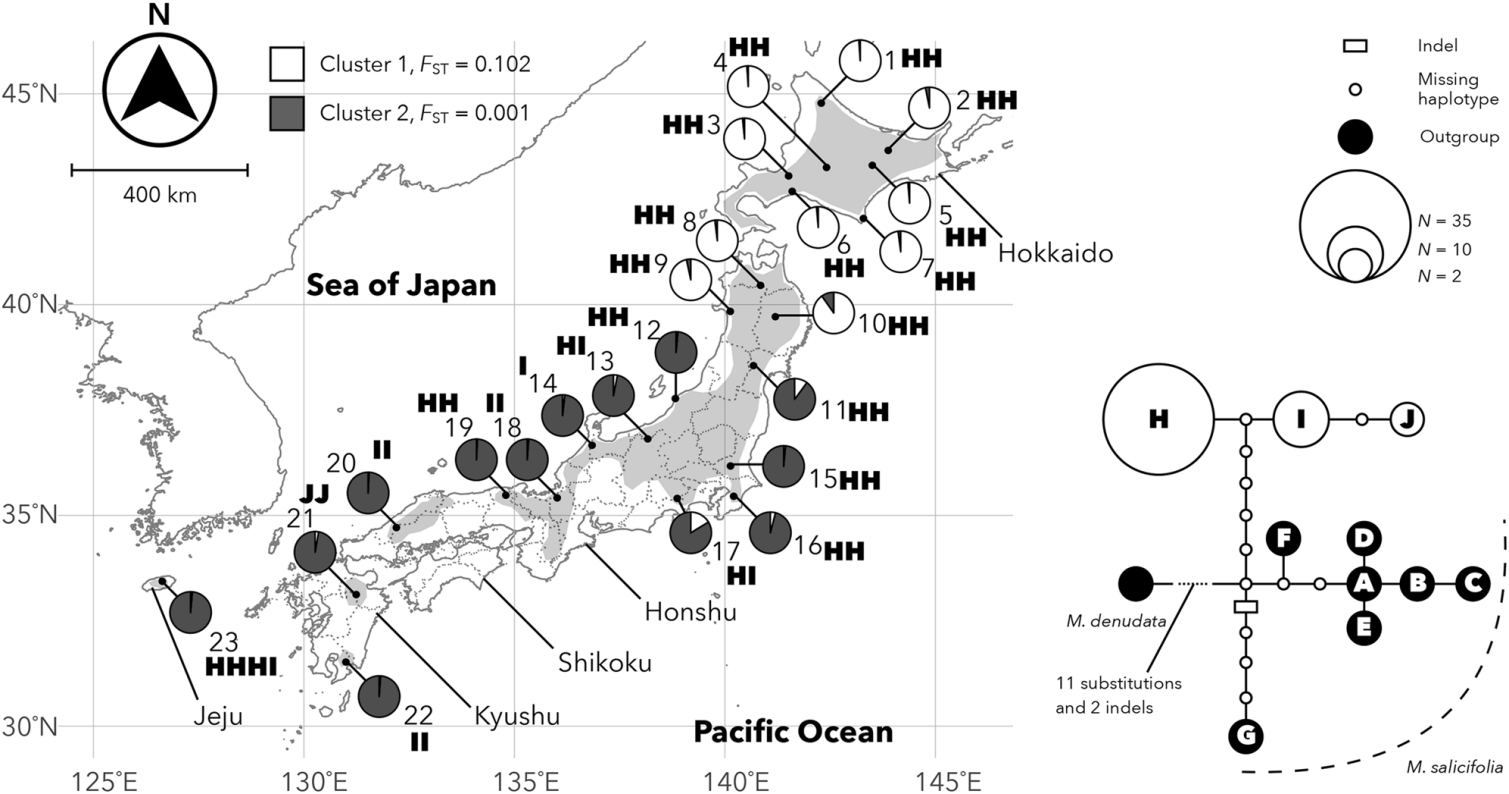
Distribution ranges of *Magnolia kobus* (gray area), the locations of the 23 populations sampled (black dots), proportions of genetic clusters detected by STRUCTURE for nuclear microsatellites (pie chart), chloroplast DNA haplotypes detected (bold type letter) and the network they formed with outgroup data [H, I and J were found in *M. kobus*, and A–G were found in its congener, *M. salicifolia* (Tamaki et al. 2018)]. Dotted lines within the Japanese archipelago indicate prefectural borders

We sampled 10–20 leaves per individual for DNA extraction and measurement of leaf morphology from 23 *Magnolia kobus* populations, which cover its entire distribution range (Fig. 1, Table 1). We sampled leaves from trees more than 20 m apart, so as not to sample leaves from the same clones, because *M. kobus* can propagate clonally by sprouting and/or layering. Two to four shoots other than water sprouts and very short shoots were cut from a sun-lit tree crown surface and the second or subsequent leaves from the top of each shoot were collected. Leaves were transported to the laboratory under cool conditions. After scanning for leaf shape, the leaves were stored at − 30 °C until required for DNA extraction. However, in population 23 (Jeju) the leaves collected were dried using silica gel and stored at room temperature prior to DNA extraction. We could not collect enough leaves for morphological measurement in this population, so the leaves were used only in genetic analysis.

**Table 1:** Location, sample size, genetic variation and leaf size of 23 *Magnolia kobus* populations

Population		Latitude	Longitude	Lineage^a^	Variety^b^	*N*_n_	*N*_c_	*N*_m_	*A*_R_	*H*_E_	*F*_IS_^c^	Leaf	
No.	Name											Length (cm)	Width (cm)
1	Nakagawa	44.78	142.29	Northern	*borealis*	14	2	14	4.02	0.700	0.074^N.S.^	12.9	6.7
2	Chimikepp lake	43.66	143.88	Northern	*borealis*	27	2	27	4.24	0.734	0.076^N.S.^	14.1	7.3
3	Nopporo	43.05	141.51	Northern	*borealis*	24	2	24	4.33	0.735	0.079^N.S.^	13.4	7.4
4	Furano	43.25	142.41	Northern	*borealis*	25	2	26	4.26	0.726	0.085*	15.3	7.7
5	Ashoro	43.30	143.50	Northern	*borealis*	7	2	8	4.06	0.731	0.098^N.S.^	14.8	7.6
6	Tomakomai	42.68	141.60	Northern	*borealis*	23	2	23	4.12	0.700	0.087*	13.2	7.2
7	Erimo	42.05	143.29	Northern	*borealis*	18	2	18	4.06	0.703	0.106*	12.3	6.5
8	Towada lake	40.45	140.84	Northern	*borealis*	21	2	21	4.11	0.708	0.081^N.S.^	12.7	6.7
9	Akita	39.84	140.12	Northern	*borealis*	12	2	12	4.54	0.758	0.096^N.S.^	13.2	7.1
10	Morioka	39.72	141.20	Northern	*borealis*	15	2	15	4.26	0.732	0.106*	12.8	7.3
11	Arasawa	38.55	140.68	Southern	*kobus*	30	2	30	4.84	0.798	0.191***	10.2	5.5
12	Kakudayama	37.77	138.82	Southern	*borealis*	22	2	22	5.02	0.832	0.012^N.S.^	13.8	7.6
13	Kurohime	36.82	138.16	Southern	*borealis*	19	2	19	4.85	0.793	0.082*	12.0	7.0
14	Kurikara	36.66	136.84	Southern	*borealis*	17	1	17	5.05	0.848	0.088*	13.9	7.4
15	Hokyosan	36.17	140.13	Southern	*kobus*	28	2	25	5.30	0.845	0.041^N.S.^	9.9	5.3
16	Nagara	35.45	140.21	Southern	*kobus*	7	2	7	4.41	0.789	0.026^N.S.^	10.1	5.3
17	Yamanaka lake	35.41	138.87	Southern	*kobus*	31	2	30	5.06	0.825	0.011^N.S.^	10.8	5.0
18	Biwa lake	35.41	136.01	Southern	*borealis*	28	2	28	4.61	0.794	0.008^N.S.^	13.5	6.8
19	Toyooka	35.48	134.80	Southern	intermediate	5	2	5	4.76	0.857	0.340***	12.2	6.5
20	Kirigaya	34.71	132.19	Southern	*kobus*	29	2	28	3.68	0.676	0.125***	12.1	5.9
21	Kujuzan	33.12	131.24	Southern	intermediate	28	2	28	5.09	0.837	0.075**	12.4	6.0
22	Otori-kyo	31.52	131.00	Southern	intermediate	7	2	7	4.98	0.859	0.245***	12.8	6.3
23	Jeju	33.43	126.63	Southern	–	16	4	–	3.34	0.580	0.146**	–	–
Average/overall													
Northern						18.6	2.0	18.8	4.20^d^	0.723^e^	0.087^f^		
Southern						20.5	2.1	20.5	4.69^d^	0.795^e^	0.081^f^		
All						19.7	2.0	19.7	4.48	0.764	0.097		

*N*_n_ number of individuals for analysis of nuclear microsatellite, *N*_c_ number of individuals for analysis of chloroplast DNA sequences, *N*_m_ number of individuals for analysis of leaf morphology, *A*_R_ allelic richness based on four diploid individuals, *H*_E_ expected heterozygosity, *F*_IS_ fixation index

^a^Lineages were determined by STRUCTURE analysis

^b^Varieties were determined by leaf morphology

^c^The significance of departure from Hardy–Weinberg equilibrium was tested by randomization test. *P* values were adjusted by Bonferroni correction. ^N.S^not significant; *P < 0.05; **P < 0.01; ***P < 0.001

^d^Southern > northern (permutation test, *P* = 0.017)

^e^Southern > northern (permutation test, *P* = 0.016)

^f^The difference in *F*_IS_ between the two lineages was not significant (permutation test)

### DNA extraction, genotyping and sequencing

Genomic DNA was extracted using a hexadecyltrimethylammonium bromide (CTAB) method (Murray and Thompson 1980) with minor modifications. Fourteen nuclear microsatellites (nSSRs) developed for *M. obovata*, M6D8 (Isagi et al. 1999), and for *M. stellata*, stm0002, stm0114, stm0163, stm0184, stm0200, stm0214, stm0223, stm0246, stm0251, stm0353, stm0383, stm0423 and stm0448 (Setsuko et al. 2005), were amplified using a Multiplex PCR Kit (QIAGEN) with a GeneAmp PCR System 9700 (Applied Biosystems) following the manufacturer’s manual. The PCR products were separated by electrophoresis on a 3100-Avant Genetic Analyzer (Applied Biosystems). Genotypes were determined using GeneMapper version 4.0 (Applied Biosystems). All genotype data were converted from fragment size to number of repeats. Before genetic analysis, in order to remove those loci with high frequencies of null alleles, we calculated the null allele frequency in each population for each locus with INEst version 1.1, which can estimate null allele frequency separately from the effect of inbreeding (Chybicki and Burczyk 2009). We calculated average null allele frequency among populations for each locus. Apart from locus stm0223, all the loci showed a null allele frequency of less than 7%, so we used the remaining 13 loci in the following analyses. Four non-coding chloroplast DNA (cpDNA) regions, *trn*S–*trn*G (Shaw et al. 2005), *trn*T–*psb*D (Shaw et al. 2007), *trn*T–*trn*L (Shaw et al. 2005; Taberlet et al. 1991) and *rpl*36–*inf*A–*rps*8–*rpl*14 (Shaw et al. 2007), were sequenced from 1 to 4 individuals in each population in the same way as described in Tamaki et al. (2018).

### Analysis of genetic diversity and differentiation

For each nSSR locus across all populations, the number of alleles (*A*), average gene diversity within population (*H*_S_), gene diversity in the total population (*H*_T_) and Weir and Cockerham’s *F*_ST_ were calculated. Hedrick’s standardized *G*_ST_ [*G*´_ST_; Hedrick (2005)] and Jost’s *D*, which is another population differentiation measure (Jost 2008), were also manually calculated. The significance of population differentiation at each locus was evaluated by a randomization test. For each population over all nSSR loci, allelic richness (*A*_R_) based on four diploid individuals, expected heterozygosity (*H*_E_) and fixation index (*F*_IS_) were calculated. The significance of departure from Hardy–Weinberg equilibrium in each population was evaluated by a permutation test. As STRUCTURE analysis detected two major genetic clusters, the 23 populations were divided into northern (populations 1–10) and the southern (11–23) lineages (see details in “Results”). Differences in *A*_R_, *H*_E_ and *F*_IS_ between the two lineages were evaluated by randomization tests. The above summary statistics except for *G*´_ST_ and *D* were calculated by FSTAT version 2.9.3.2 (Goudet 1995). The presence of an isolation by distance pattern, which is a significant correlation between geographic and genetic distances, was investigated by the Mantel test with R package ade4 version 1.7.11 (Chessel et al. 2004). Kilometers on the log scale and *F*_ST_/(1 − *F*_ST_) between population pairs were used as geographic and genetic distances, respectively. Population-based principal component analysis using allele frequency data was conducted using R package ade4.

Genetic structure among populations was investigated with the model based clustering method implemented in STRUCTURE version 2.3.4 (Falush et al. 2003; Pritchard et al. 2000). The admixture and correlated allele frequency models were used. As suggested by Wang (2017), different *α* values for each genetic cluster were estimated and a low initial value of *α* = 0.05 was applied. Different numbers of genetic clusters (*K*) from 1 to 16 were tested. The first 40,000 iterations were discarded as a burn-in period and then 40,000 iterations were used for the estimation of membership of each genetic cluster for each individual. The estimations of parameters were repeated 10 times for each *K*. CLUMPAK was used to check the multimodality within the same *K* (Kopelman et al. 2015). LargeKGreedy option was selected and the number of repeats was set to 500. To estimate the optimal *K*, the log probability of data and *ΔK* for each *K* were estimated with R package corrsieve version 1.6.8 (Campana et al. 2011; Evanno et al. 2005). Analysis of molecular variance (AMOVA) was performed with Arlequin version 3.5.2 (Excoffier and Lischer 2010). Genetic variation was hierarchically divided into three layers, which were the lineages inferred by STRUCTURE analysis, populations and individuals, and variance components for each layer and related *Φ*-statistics were calculated. The significance of each *Φ*-statistic was evaluated by the permutation test implemented in Arlequin.

CpDNA sequences were edited and assembled with DNA baser version 3 (Heracle BioSoft SRL), and then aligned with the MUSCLE algorithm in MEGA version 5.1 (Edgar 2004; Tamura et al. 2011). Mono- or di-nucleotide repeats in the sequences were omitted from subsequent analysis to avoid the possibility of homoplasy. CpDNA haplotypes were determined and a network among them was constructed using TCS version 1.21 (Clement et al. 2000). The number of polymorphic sites and the mean number of pairwise differences were calculated, and Tajima’s test for selective neutrality (Tajima 1989) was performed with Arlequin.

### Analysis of variation in leaf morphology

We used 9.8 leaves on average per individual tree, and a total of 4,260 leaves for analysis of leaf morphology. The length and width of each leaf were measured and their average values in each individual were calculated. Numerical conversion of leaf shape into elliptic Fourier descriptors and measurement of leaf area were conducted with SHAPE version 1.3 (Iwata and Ukai 2002). Principal component analysis of leaf shape variables was conducted by SHAPE. Principal components (PCs) whose contribution to the total variance of data was more than 5% (PC1, PC2 and PC3) were used (see details in “Results”). Because PC3 represented the asymmetry of leaf shape and positive and negative values therefore probably had no biological meaning, and also to ensure normality, log-transformed absolute values of PC3 were used.

To evaluate the effects of environmental factors and population history on leaf morphological traits, a Bayesian linear mixed model was constructed. For response variables, PC1, PC2, PC3 and leaf area were used. In this analysis, we just intended to evaluate whether environmental factors affected leaf morphological traits or not and did not intend to specify what environmental factors affected them. Thus, all 19 bioclimatic variables were downloaded from WorldClim (http://www.worldclim.com) and principal component analysis with the data for the variables was conducted using prncomp function of R. Principal components (BioPCs) estimated by 19 bioclimatic variables were used as explanatory variables for environmental effects. Only BioPCs whose contribution to the total variance of data were more than 5% (BioPC1, BioPC2, BioPC3 and BioPC4) were used (see details in “Results”). Membership coefficient of the northern lineage estimated by STRUCTURE at *K* = 2 (Q) was also used as an explanatory variable for population history. In the preliminary analysis, we calculated correlation coefficients between BioPCs and Q. Because a correlation coefficient between BioPC1 and Q was − 0.823, we removed BioPC1 from the following analysis. On the other hand, absolute values of the other correlation coefficients were less than 0.213. Thus, finally, we used BioPC2, BioPC3, BioPC4 and Q for explanatory variables. Replicates within an individual were treated as a random effect. Constructed Bayesian linear mixed model was as follows:

$$\begin{aligned} & Y_{\text{Exp}} \left[ i \right]\, = \,\beta_{0} \left[ j \right]\, + \,\beta_{\text{BioPC2}} \, \times \,{\text{BioPC2}}\left[ i \right]\, + \,\beta_{\text{BioPC3}} \, \times \,{\text{BioPC3}}\left[ i \right]\, + \,\beta_{\text{BioPC4}} \, \times \,{\text{BioPC4}}\left[ i \right]\, + \,\beta_{\text{Q}} \, \times \,{\text{Q}}\left[ i \right] \\ & {\text{Y}}_{\text{Obs}} \left[ i \right]\,\sim \,{\text{Normal }}\left( {Y_{\text{Exp}} \left[ i \right],\sigma_{\text{All}} } \right) \\ & \beta_{0} \left[ j \right]\,\sim \,{\text{Normal }}\left( {\beta_{{0\_{\text{Mean}}}} ,\sigma_{\text{Individual}} } \right) \\ \end{aligned}$$

Italic and roman characters indicate the parameters to be estimated and observed values, respectively. Indices *i* and *j* are leaf and individual IDs, respectively. *Y*_Exp_ and Y_Obs_ are expected and observed values of response variables, respectively. *β*_0_[*j*] is an intercept and the other *β*s are regression coefficients. *σ*s are standard deviations and assumed to take a value greater than zero. Non-informative priors were used for all parameters. Stan via rstan package version 2.18.2 of R was used to estimate posterior distributions of parameters (Stan Development Team 2018). Four independent Markov chains were run. Each Markov chain was constructed 4,000 iterations and the first 1,000 iterations were discarded as a burn-in period. Posterior distributions were sampled by 10 steps and, finally, 300 × 4 = 1,200 samples were used to estimate the posterior mode and 95% highest posterior density (HPD). The posterior mode was estimated using the density function of R. The 95% HPD was estimated using coda package version 0.19.1 in R (Plummer et al. 2006). We considered *β*s significant if the 95% HPD did not overlap zero.

### Inference of population demography

To infer the population demography of the two distinct lineages, the northern and southern lineages, detected by STRUCTURE analysis, an approximate Bayesian computation (ABC) approach was applied. We used only pure populations without admixture from the recessive lineage at *K* = 2 (less than 5%) and populations 10, 11 and 17 were thus removed from this analysis. To reduce the computational cost and uncertainty of parameter estimation, the parameters of the demographic model were sequentially inferred (Chen et al. 2017; Hiraoka et al. 2018; Tamaki et al. 2018). First, population size change models were applied for each lineage and then models of population divergence between the two lineages were applied using information obtained from the population size change model. We used 13 nSSR and 3,929 bp cpDNA sequences, from which insertions/deletions (indels) were removed. In population size change analysis, the average and standard deviation of the number of alleles, heterozygosity and allele size range for nSSRs, and the number of polymorphic sites and mean number of pairwise nucleotide differences for cpDNA sequences, were calculated. A total of eight summary statistics were used. In population divergence analysis, in addition to the same summary statistics as for population size change analysis, allele size range for samples overall, *F*_ST_ of overall loci for nSSR and cpDNA sequences were calculated. A total of 19 summary statistics were used. The software arlsumstat version 3.5.2 was used for calculation of summary statistics (Excoffier and Lischer 2010).

**Fig. 2:**
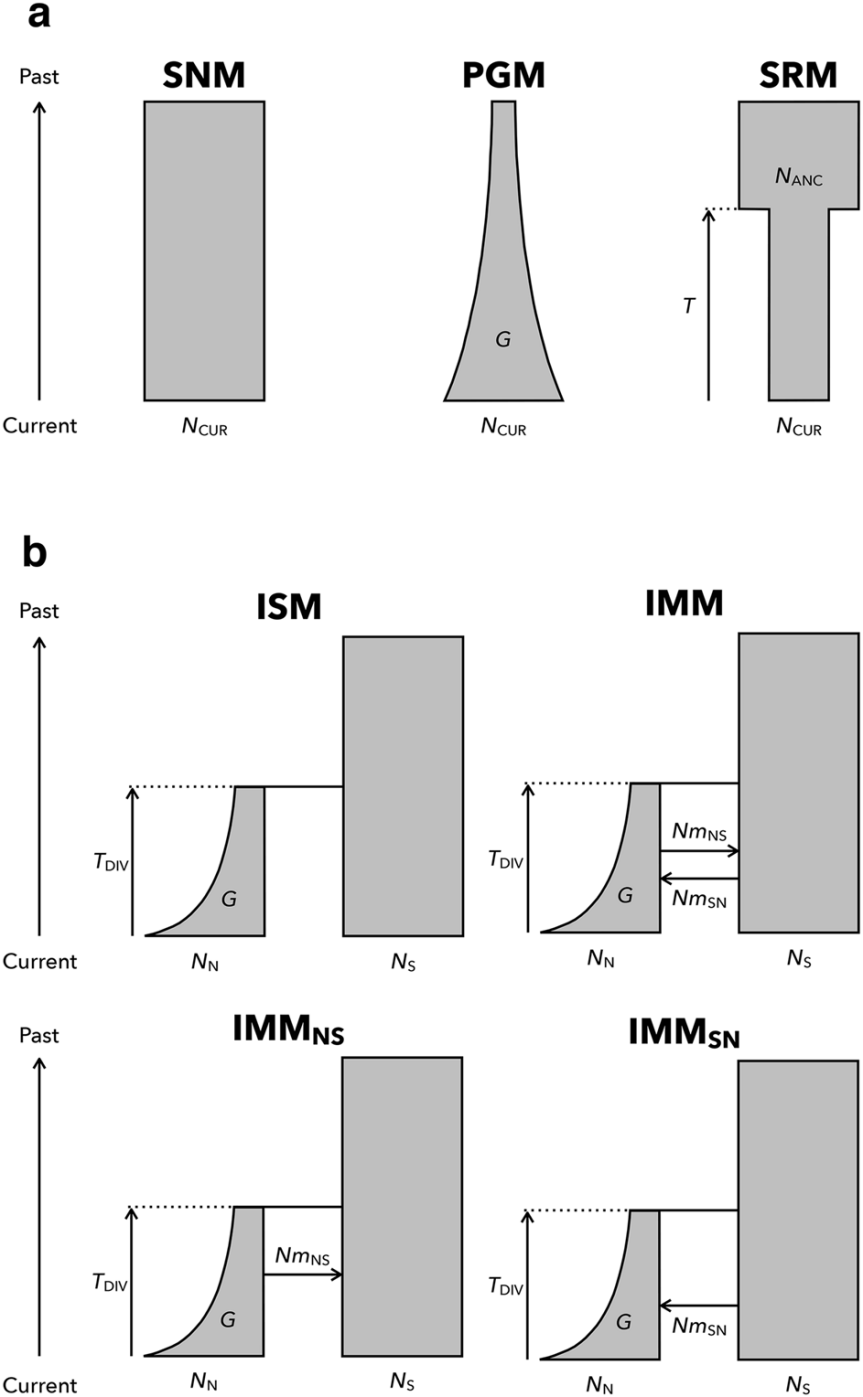
Comparison of three population size change (**a**) and four population divergence (**b**) models. *SNM* standard neutral model, *PGM* population growth model, *SRM* size reduction model, *ISM* isolation model, *IMM* isolation with migration model, *IMM*_*NS*_ model of isolation with one way migration from the northern to the southern lineages, *IMM*_*SN*_ model of isolation with one way migration from the southern to the northern lineages. Direction of migration is backward-in-time

Three distinct population size change models were built (Fig. 2a). (1) The standard neutral model (SNM) assumed that the effective population size was constant from the current to the past and had one structural parameter, current effective population size (*N*_CUR_). The unit of *N*_CUR_ was the number of diploid individuals. (2) the population growth model (PGM) assumed that the current effective population size shrank exponentially towards the past with the rate *G* [*Nt* = *N*_CUR_ × exp (*G* × *t*); *Nt* was the effective population size at time *t*], i.e. there was exponential growth from past to present. PGM had two structural parameters, *N*_CUR_ and *G*. The unit of the time parameter was generations. (3) The size reduction model (SRM) assumed that the effective population size changed at time *T* and had three structural parameters *N*_CUR_, *T* and relative ancestral effective population size [*RN*_ANC_; *RN*_ANC_ > 1 and ancestral population size (*N*_ANC_) = *N*_CUR_ × *RN*_ANC_]. Prior distributions of parameters of the three models are shown in Table S1. We simulated nSSR and cpDNA sequence data simultaneously under these three models by fastsimcoal2 version 2.5.2.21 (Excoffier and Foll 2011). As fastsimcoal2 used the number of gene copies as the unit of effective population size, twice the value of *N*_CUR_ was passed to it when we simulated the nSSR data. On the other hand, the raw value of *N*_CUR_ was passed to it when we simulated the cpDNA sequence data because *M. kobus* is monoecious. A generalized stepwise mutation model (GSM) was used as a mutation model for nSSRs (Estoup et al. 2002). GSM has two parameters, mutation rate per generation (*μ*) and the geometric parameter (*P*_GSM_). *P*_GSM_ ranges from 0 to 1 and represents the proportion of mutations that change allele sizes by more than one step; a value of zero means a strict stepwise mutation model. We simulated 13 independent loci. The prior distribution for the mean value of *μ* among 13 loci was drawn from a log-uniform distribution from 10^−5^ to 10^−3^ and each locus value of *μ* was randomly drawn from a gamma distribution with *shape* and *rate* parameters. The prior distribution of the *shape* parameter was drawn from a uniform distribution from 0.5 to 5 and the *rate* parameter was calculated by *shape*/the mean value of *μ*. The prior distribution of the mean value of *P*_GSM_ among the 13 loci was drawn from a uniform distribution from 0 to 1 and each locus value of *P*_GSM_ was randomly drawn from a beta distribution with *a* and *b* parameters. The values of *a* and *b* were calculated by 0.5 + 199 × the mean value of *P*_GSM_ and *a* × (1 − the mean value of *P*_GSM_)/the mean value of *P*_GSM_, respectively, according to Excoffier et al. (2005). For cpDNA sequences, we simulated 3,929 bp sequences and the value of mutation rate for cpDNA was set to 2.0 × 10^−9^ substitutions per site per generation (Muse 2000; Sakaguchi et al. 2012).

All priors were generated using R version 3.5.0 (R Core Team 2018). The three size change models were simulated 10,000 times and summary statistics were calculated by arlsumstat. The three models were compared using the ABC random forest (ABC-RF) approach implemented in abcrf package version 1.7 of R (Pudlo et al. 2016). ABC-RF can yield similar results to those of the conventional ABC with much smaller numbers of simulations and thus greatly reduce the computational time required. The number of trees in the random forest was set to 1,000. The classification error rate and posterior probability of the best model were calculated. For the best model, 5 × 10^5^ simulations were conducted and posterior distributions of parameters were estimated using neural network regression implemented in abc package version 2.1 in R with the logit transformation option (Csillery et al. 2012). The tolerance value was set to 0.005 and 2,500 and summary statistics nearest to the observed data were used for the parameter estimation. The posterior mode and 95% HPD were estimated in the same way as Bayesian linear mixed model analysis.

Taking into account the results of the population size change models, four different population divergence models were constructed (Fig. 2b). (1) The isolation model (ISM) assumed that the two lineages diverged at time *T*_DIV_ and had four structural parameters, *N*_N_, *N*_S_, *T*_DIV_ and *G*. *N*_N_ and *N*_S_ were the effective population sizes of the northern and the southern lineage, respectively. The value of *G* was fixed at − 1.56 × 10^−4^ using the value of the mode estimated in the population size change analysis in order to reduce the computational costs (see details in “Results”). (2) The isolation with migration model (IMM) assumed that there were migrations between lineages after divergence and it had six structural parameters, *N*_N_, *N*_S_, *T*_DIV_, *G* and numbers of migrants per generation from the northern to the southern and from the southern to the northern lineage in the backward-in-time direction (*Nm*_NS_ and *Nm*_SN_, respectively). When running simulations, *Nm*_NS_ and *Nm*_SN_ were divided by *N*_N_ and *N*_S_, respectively, then the migration rates calculated were passed to fastsimcoal2. In angiosperms, the migration rate measured in the nuclear genome reflects both pollen and seed dispersals, whereas that in the chloroplast genome reflects only seed dispersal because the chloroplast genome is generally maternally-transmitted. When simulating cpDNA sequences, we thus multiplied migration rates by a coefficient *β*, which ranges from 0 to 1, in order to take account of the reduction in the migration rate for the chloroplast genome. The prior distribution of *β* was drawn from a uniform distribution from 0 to 1. (3) and (4) IMM models with one way migration from the northern to the southern lineage (IMM_NS_) and from the southern to the northern lineage (IMM_SN_) were also defined. For the mutation parameters of the four population divergence models, the same settings as for population size change analysis were used. All prior distributions for parameters are listed in Table S1. Model choice and parameter estimation of the best model were conducted in the same way as population size change analysis except for the number of simulations (1.5 × 10^6^) and tolerance value (0.002; 3,000 summary statistics nearest to the observed data) when parameter estimation was carried out.

To confirm that the model fits the observed data, posterior predictive simulations using 1,000 randomly drawn posterior samples were conducted for both population size change and population divergence analyses (Gelman et al. 2014). Summary statistics were calculated and compared to the observed data.

### Ecological niche modeling

Ecological niche modeling was performed to infer the possible distribution ranges of *M. kobus* in the last glacial maximum (LGM; 21 kya) and last inter-glacial (LIG; 130 kya) with the maximum entropy method implemented in Maxent version 3.3.3 k (Phillips et al. 2006). We used 101 location data for sites where the occurrence of *M. kobus* was recorded. These location data consisted of the 23 populations sampled in this study, our field observations and records from the Global Biodiversity Information Facility [GBIF.org (16 April 2017) GBIF Occurrence Download http://doi.org/10.15468/dl.ifurvo]. All records from GBIF were carefully checked against satellite images on Google Maps (http://maps.google.com) and ambiguous or erroneous location data were removed. A current distribution model was constructed with six bioclimatic variables, annual mean temperature (bio1), mean temperature of warmest quarter (bio10), mean temperature of coldest quarter (bio11), annual precipitation (bio12), precipitation in warmest quarter (bio18) and precipitation in coldest quarter (bio19), at a resolution of 2.5 arc-minutes, as used in a study of *Magnolia salicifolia* (Tamaki et al. 2018), a species growing in a similar climate zone. Validation of the model was performed using 100 replicates of cross-validation procedures, with 25% of the data for model testing, implemented in Maxent. Assuming temporal stability of ecological niche for *M. kobus*, the model constructed was applied to LGM and LIG climatic layers to predict the past distributions of the species. The model for interdisciplinary research on climate [MIROC; Hasumi and Emori (2004)] and the community climate system model [CCSM; Collins et al. (2006)] were used to predict the distributions during the LGM. All data for bioclimatic variables used in this modelling were obtained from WorldClim. To determine the coastal line at the LGM, we obtained the ETOPO1 Global Relief Model (10.7289/V5C8276M) and only predicted areas higher than − 130 m from the present level were clipped out.

## Results

### Genetic diversity and differentiation

Among the 13 nuclear microsatellite loci across 23 populations, the number of alleles (*A*) ranged from 12 to 37 with an average value of 24.6 and the average gene diversity within populations (*H*_S_) ranged from 0.388 to 0.911 with an average value of 0.762 (Table 2). The values of *F*_ST_, *G*´_ST_ and Jost’s *D* over the 13 loci were 0.119, 0.504 and 0.439, respectively. All of the 13 loci showed significant population differentiation. Among the 23 populations over the 13 loci, allelic richness (*A*_R_) based on four individuals ranged from 3.34 to 5.30 with an average value of 4.48 and expected heterozygosity (*H*_E_) ranged from 0.580 to 0.859 with an average value of 0.764 (Table 1). Fixation index (*F*_IS_) ranged from 0.008 to 0.340 within populations and its value over all populations was 0.097. Twelve of the 23 populations showed significant deviation from Hardy–Weinberg equilibrium.

**Table 2:** Genetic diversity at 13 nuclear microsatellite loci across 23 *Magnolia kobus* populations

Locus	*A*	*H*_S_	*H*_T_	*F*_ST_	*G*´_ST_	*D*
M6D8^a^	24	0.796	0.917	0.125	0.670	0.620
stm0002^b^	19	0.761	0.892	0.154	0.636	0.573
stm0114^b^	17	0.388	0.585	0.350	0.559	0.337
stm0163^b^	21	0.823	0.893	0.080	0.457	0.413
stm0184^b^	30	0.839	0.924	0.097	0.593	0.552
stm0200^b^	33	0.781	0.828	0.062	0.265	0.224
stm0214^b^	19	0.740	0.867	0.150	0.580	0.511
stm0246^b^	34	0.911	0.952	0.045	0.503	0.482
stm0251^b^	16	0.642	0.738	0.125	0.377	0.280
stm0353^b^	22	0.860	0.927	0.076	0.534	0.500
stm0383^b^	36	0.880	0.936	0.059	0.520	0.488
stm0423^b^	37	0.815	0.935	0.134	0.718	0.678
stm0448^b^	12	0.672	0.813	0.185	0.547	0.449
Average/overall	24.6	0.762	0.862	0.119	0.504	0.439

*A* number of alleles, *H*_S_ average gene diversity within populations, *H*_T_ gene diversity in the total population, *F*_ST_ Weir & Cockerham’s *F*_ST_, *G′*_ST_ Hedrick’s standardized *G*_ST_, *D* Jost’s *D*

^a^Isagi et al. (1999)

^b^Setsuko et al. (2005)

**Fig. 3:**
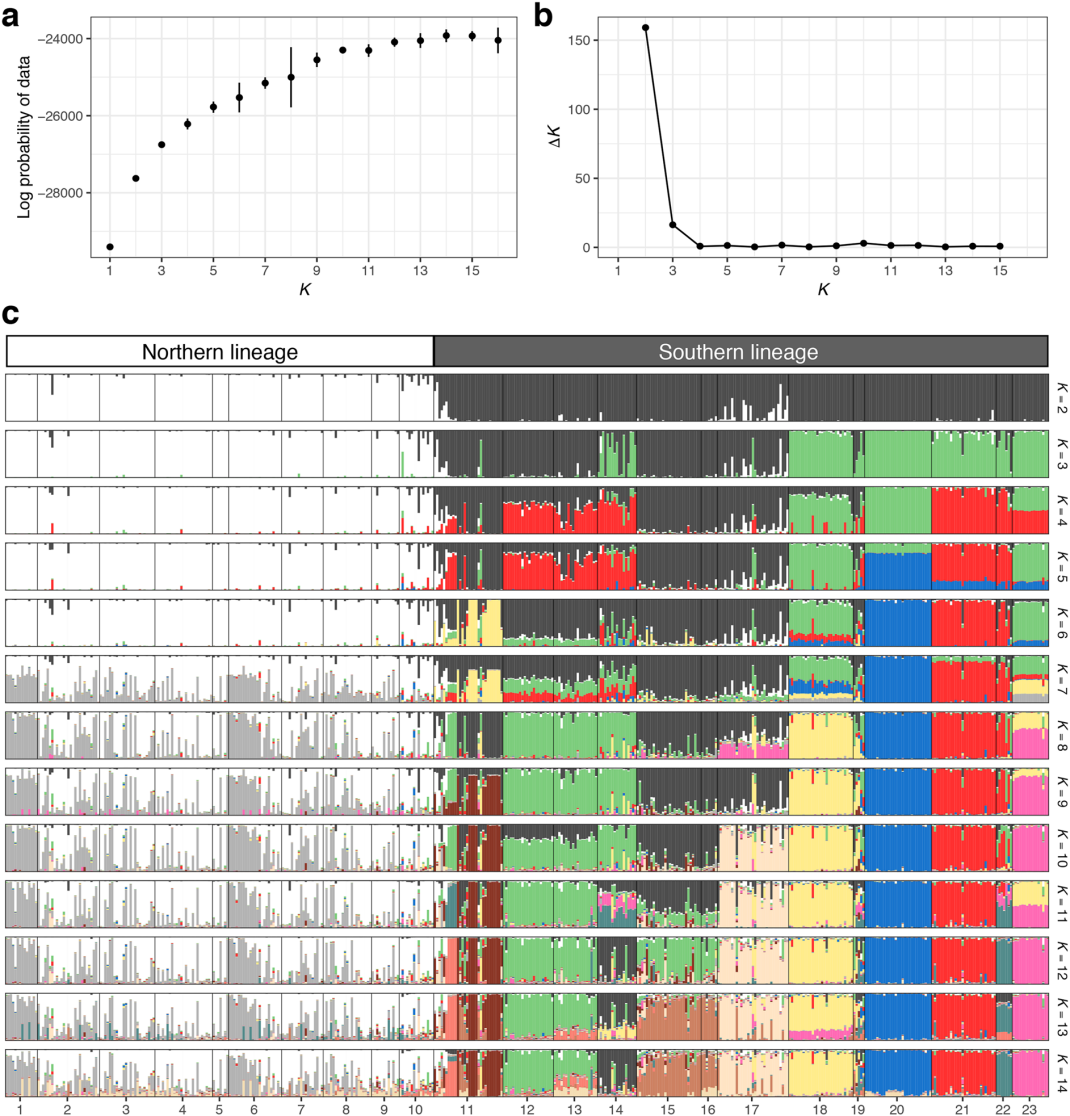
Changes in log probability of data (**a**) and *ΔK* (**b**) along the number of genetic clusters (*K*) in STRUCTURE analysis of 453 *Magnolia kobus* individuals sampled from 23 populations. Distributions of genetic clusters in each individual from *K* = 2 to 14 (**c**)

The log probability of data in each *K* estimated by STRUCTURE analysis increased with increasing *K* until it reached a plateau at *K* = 14 (Fig. 3a). However, *ΔK* was highest at *K* = 2 (Fig. 3b). We therefore considered that *K* = 2 and 14 were the optimal *K*s. By using CLUMPAK, we checked the multimodality within the same *K* from *K* = 2 to 14 and found multiple modes except when *K* = 2 and 7. We carefully checked changes in cluster distribution along *K*, determined appropriate cluster distribution at each *K* by basically choosing major modes except when *K* = 8 and 12, and constructed a series of barplots for membership coefficients (Fig. 3c). The distribution of genetic clusters at *K* = 2 showed clear separation between the northern and southern regions (Figs. 1, 3c). Accordingly, we classified the 23 populations into northern (populations 1–10) and southern lineages (11–23). The northern and southern lineages were dominated by clusters 1 and 2, respectively. The value of *F*_ST_ between each cluster and the ancestral population was ca. 100 times greater for cluster 1 (0.102) than for cluster 2 (0.001). Populations 10, 11 and 17 showed more than 5% genetic admixture of the recessive cluster. At *K* = 14, although populations 1–10 were dominated by the mixture of two clusters, most of the remaining 13 populations were dominated by one of the other 12 clusters (Fig. 3c). The genetic clusters that dominated in populations 11, 20 and 23 showed larger values of *F*_ST_ between each cluster and the ancestral population (> 0.33).

Population based principal component analysis also showed that populations in the northern lineage aggregated on the principal component axes (Fig. S1). Populations 20 and 23 were located apart from the other populations on the axes. *A*_R_ and *H*_E_ were higher in the southern lineage than in the northern lineage with the exceptions of populations 20 and 23 (Table 1, Fig. S2). Average values of *A*_R_ and *H*_E_ in the southern lineage were significantly higher than those in the northern one (permutation test, *P* = 0.017 and 0.016 for *A*_R_ and *H*_E_, respectively; Table 1). The difference in *F*_IS_ between the two lineages was not significant.

Significant isolation by distance (IBD) patterns were detected over all 23 populations (*R*^2^ = 0.400 and *P* < 0.001) and in both the northern and the southern lineages (*R*^2^ = 0.150 and 0.263, and *P* = 0.009 and 0.003, respectively; Fig. S3). However, the strength of IBD in the northern lineage was less than that in the southern one.

A total of 3,932 bp-length of aligned cpDNA sequences was obtained. Four substitutions were detected within the species and three haplotypes (H, I and J) were determined (Fig. 1, Table S2). Although only haplotype H was detected in the northern lineage, all three haplotypes were detected in the southern one. In the southern lineage, the number of polymorphic sites, mean number of pairwise differences and Tajima’s *D* were 4, 1.311 and 0.695, respectively. The result of Tajima’s test for selective neutrality was not significant. All of the three haplotype sequences were deposited in the DDBJ/EMBL/GenBank database (LC421491–LC421502).

AMOVA was performed with three layers, between lineages, among populations within lineages and among individuals within populations (Table 3). Both nSSR and cpDNA haplotypes showed significant divergence between lineages with *Φ*_CT_ values of 0.058 and 0.308, respectively.

**Table 3:** Results from analysis of molecular variance for nuclear microsatellites and chloroplast DNA haplotypes

Layer	Nuclear microsatellites		Chloroplast DNA haplotypes	
	Variance component (%)	*Φ*-statistics	Variance component (%)	*Φ*-statistics
Between lineages	5.8	*Φ*_CT_ = 0.058***	30.8	*Φ*_CT_ = 0.308**
Among populations within lineages	8.8	*Φ*_SC_ = 0.094***	43.4	*Φ*_SC_ = 0.627**
Among individuals within populations	85.4	*Φ*_ST_ = 0.146***	25.8	*Φ*_ST_ = 0.741***

**P < 0.01, ***P < 0.001

### Leaf morphological variation

**Fig. 4:**
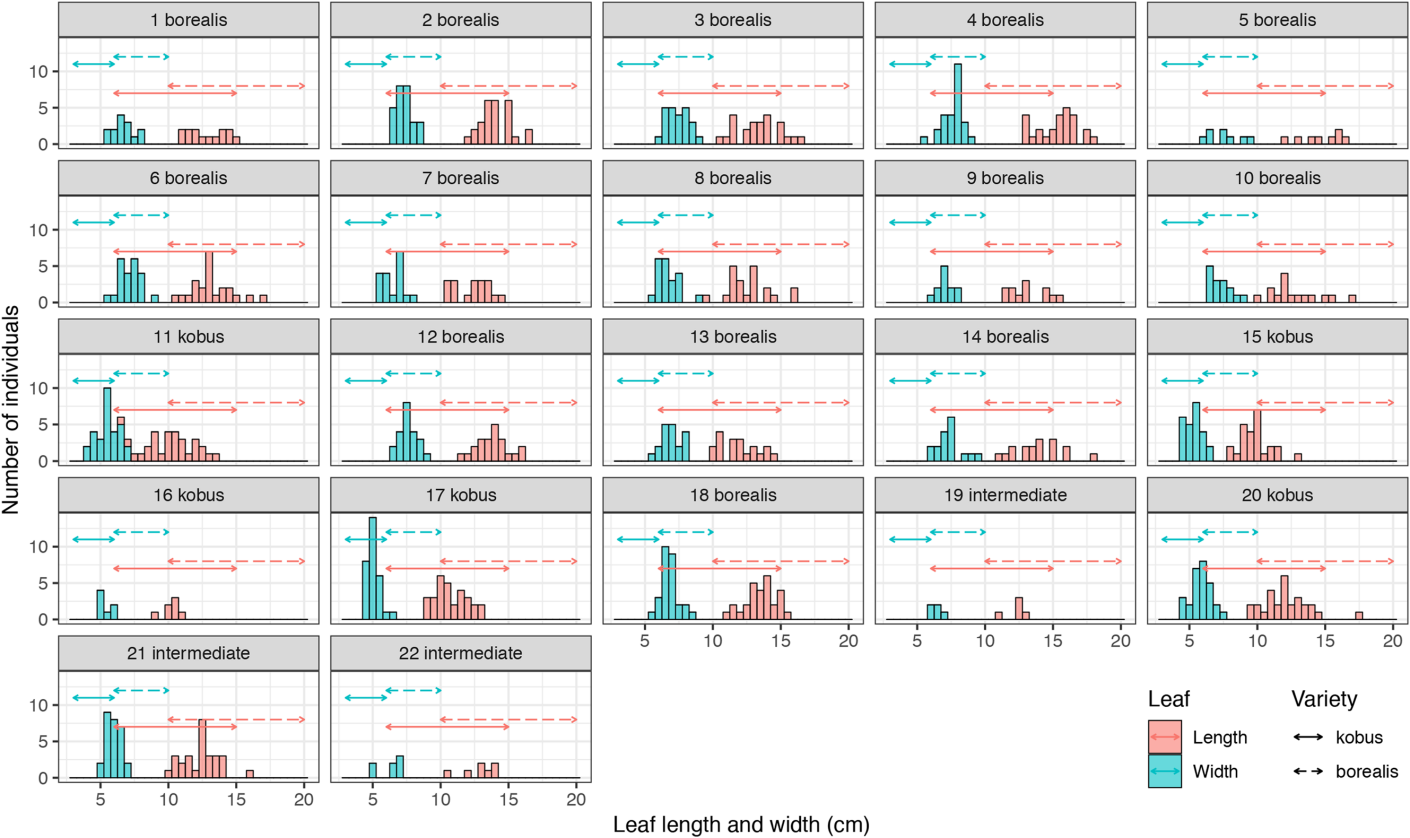
Distributions of average values of leaf length and width within trees for 22 *Magnolia kobus* populations (the Jeju population, No. 23, was excluded from this analysis). Arrows indicate the ranges of leaf length and width for varieties *kobus* and *borealis* according to Ohashi (2015). The population numbers (see Table 1) and variety names are shown on each panel. We classified populations into two varieties based on the distributions of average values of leaf length and width. “intermediate” indicates that we could not determine varieties because the distributions were intermediate between those for two varieties

**Fig. 5:**
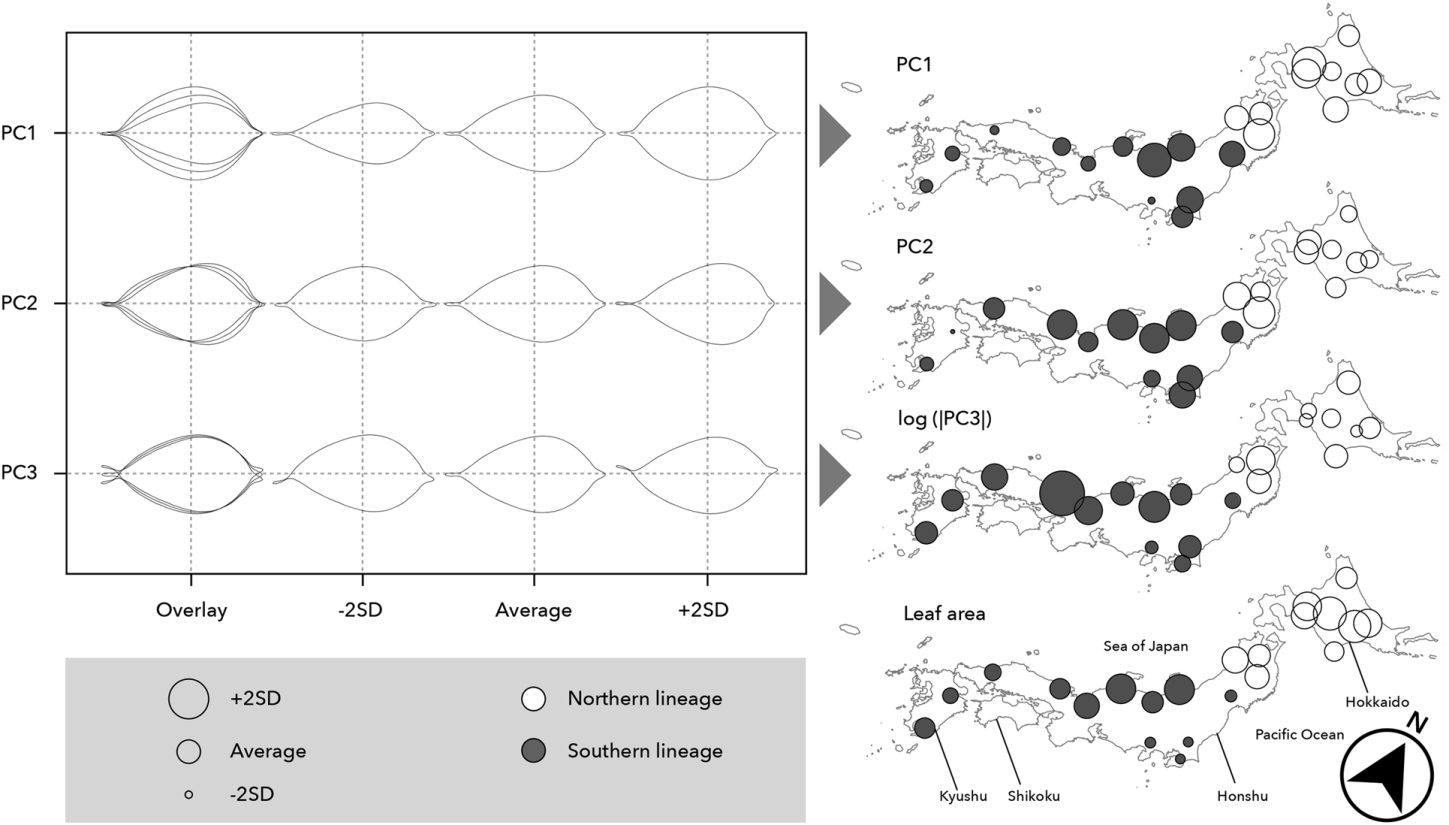
Geographical changes in leaf shape and area for 22 *Magnolia kobus* populations (the Jeju population, No. 23, was excluded from this analysis). Leaf shape was extracted with an elliptic Fourier method and converted into principal components (PCs) by SHAPE. Only PCs whose contribution to the overall variance was more than 5% are shown

Based on the definitions of varieties *kobus* and *borealis* by Ohashi (2015) and the distributions of average values of leaf length and width within trees in each population, we classified populations into two varieties (Table 1, Fig. 4). Populations 1–10, 12–14, and 18 were classified into variety *borealis*. Populations 11, 15–17 and 20 were classified into variety *kobus*. However, because the distributions of average values of leaf width and length for populations 19, 21 and 22 fell into the boundary between the two varieties, we could not determine varieties for these three populations. Moreover, we examined three principal components (PCs) estimated by SHAPE which had more than 5% contribution to the overall variance (Table S3). PC1 reflected relative leaf width, for which populations in central to northern Honshu Island and Hokkaido Island had large values (Fig. 5). PC2 reflected the position of the maximum width and populations in Kyushu Island had small values. PC3 reflected leaf curvature, with positive and negative values indicating left and right, respectively. As symmetry of curvature had no ecological meanings, the values of PC3 were log-absolute transformed and they were shown on the map. However, no geographical tendencies were observed. Populations located on the Sea of Japan side from central to northern Japan and on Hokkaido Island had large leaf area values. The populations classified as variety *borealis* showed large values in both PC1 and leaf area.

Four BioPCs had more than 5% contribution to the overall variance (Fig. S4). All explanatory variables (BioPC2, BioPC3, BioPC4 and Q) were significant in the Bayesian linear mixed effect models of PC1 and leaf area (Table 4). Only two explanatory variables related to environmental factors (BioPC2 and BioPC3) were significant in the model of PC2. However, no explanatory variables in the model of PC3 were significant.

**Table 4:** Posterior mode (95% highest posterior density) of parameters for the Bayesian linear mixed effect model explaining leaf morphological traits for *Magnolia kobus*

Parameter	PC1	PC2	log (|PC3|)	Leaf area
*β*_0_Mean_	**−0.0086 (−0.0145** to **−0.0024)**	0.0013 (−0.0019 to 0.0039)	**−4.176 (−4.227** to **−4.106)**	**46.40 (44.73** to **47.97)**
*β*_BioPC2_	**−0.0042 (−0.0067** to **−0.0021)**	**−0.0057 (−0.0068** to **−0.0046)**	−0.0294 (−0.0494 to 0.0006)	**−3.814 (−4.362** to **−3.078)**
*β*_BioPC3_	**0.0030 (0.0005** to **0.0055)**	**0.0031 (0.0019** to **0.0042)**	−0.0206 (−0.0379 to 0.0116)	**−3.133 (−3.805** to **−2.529)**
*β*_BioPC4_	**−0.0066 (−0.0107** to **−0.0007)**	−0.0011 (−0.0030 to 0.0013)	−0.0027 (−0.0487 to 0.0455)	**3.283 (2.102** to **4.686)**
*β*_Q_	**0.0223 (0.0131** to **0.0317)**	−0.0004 (−0.0047 to 0.0043)	−0.0843 (−0.1956 to 0.0051)	**13.06 (10.80** to **16.00)**
*σ*_Individual_	0.0448 (0.0415 to 0.0479)	0.0204 (0.0188 to 0.0219)	0.308 (0.269 to 0.362)	11.61 (10.80 to 12.59)
*σ*_All_	0.0406 (0.0398 to 0.0417)	0.0227 (0.0222 to 0.0232)	1.059 (1.036 to 1.082)	13.10 (12.80 to 13.38)

Leaf shape was extracted with an elliptic Fourier method and converted into principal components (PCs) by SHAPE. *β*_0_Mean_ is the average intercept among individuals. The other *β*s are regression coefficients. *β*s that were significantly deviated from 0 are shown in bold. *σ*s are standard deviation parameters of a normal distribution in the model

*BioPC* principal component estimated by 19 bioclimatic variables, *Q* membership coefficient of the northern lineage estimated by STRUCTURE at *K* = 2

### Population demography

In the model choice among the three population size change models, the population growth model (PGM) and the standard neutral model (SNM) were selected as the best models for, respectively, the northern and the southern lineages, with high posterior probabilities of 0.973 and 0.837, and low classification error rates of 0.193 and 0.192 (Table 5). All posterior distributions of the parameters of the best models showed clear single peaks (Fig. S5, Table 6).

**Table 5:** Classification error rate, proportion of votes by random forest (RF) composed of 1,000 trees based on a trained set of 10,000 simulations, best model (shown in bold) selected by RF and its posterior probability

Analysis	Lineage	Classification error rate	Proportion of votes by RF							Posterior probability
			SNM	PGM	SRM	ISM	IMM	IMM_NS_	IMM_SN_	
Population size change	Northern	0.193	0.032	**0.962**	0.006	–	–	–	–	0.973
	Southern	0.192	**0.852**	0.012	0.136	–	–	–	–	0.837
Population divergence		0.323	–	–	–	**0.609**	0.019	0.043	0.329	0.842

**Table 6:** Posterior mode (95% highest posterior density) of parameters for population size change and population divergence models

Lineage	Population size change		Population divergence
	Northern	Southern	
Best model	PGM	SNM	ISM
*N*_CUR_ (× 10^4^)	0.95 (0.18 to 10.17)	5.63 (2.91 to 14.49)	–
*G* (× 10^−4^)	− 1.56 (− 9.36 to − 0.24)	–	Fixed to − 1.56
*N*_N_ (× 10^4^)	–	–	7.37 (3.01 to 14.20)
*N*_S_ (× 10^4^)	–	–	12.03 (6.97 to 14.91)
*T*_DIV_ (× 10^4^)	–	–	1.13 (0.47 to 3.20)
Mean *μ* (× 10^−4^)	2.35 (0.45 to 8.53)	0.90 (0.40 to 3.37)	0.59 (0.27 to 1.83)
*shape*	1.90 (0.73 to 4.23)	2.48 (1.21 to 4.89)	1.52 (0.68 to 4.04)
Mean *P*_GSM_	0.585 (0.488 to 0.664)	0.421 (0.192 to 0.554)	0.440 (0.237 to 0.571)

Direction of migration is backward-in-time. The unit of effective population size is the number of diploid individuals. A negative value of *G* indicates exponential population growth from the past to the present. The unit of *T*_DIV_ is generations ago

*PGM* population growth model, *SNM* standard neutral model, *ISM* isolation model, *N*_CUR_ current effective population size, *G* population growth rate, *N*_N_*and N*_S_ current effective population size of the northern and southern lineages, respectively, *T*_DIV_ divergence time, *mean μ, shape and mean P*_GSM_ parameters of mutation model for nuclear microsatellites

Using the information obtained from population size change analyses, we constructed four population divergence models with different migration patterns. Model choice among the models was carried out and the isolation model (ISM) was selected as the best model with posterior probability 0.842 (Table 5). Although the classification error rate for population divergence analysis was a little high (0.323), since the probabilities that the other models were wrongly assigned to ISM were very low (from 0.005 to 0.068; Table S4), we concluded that the correct model was selected by ABC-RF. All posterior distributions of the best model showed clear single peaks (Fig. S6). Estimated posterior modes (95% HPD) of current effective population sizes for the northern and southern lineages (*N*_N_ and *N*_S_) were 73,700 (30,100–142,000) and 120,300 (69,700–149,100), respectively (Table 6). The values were similar between the two lineages. The posterior mode (95% HPD) of divergence time (*T*_DIV_) was 11,300 (4,700–32,000) generations ago.

Posterior predictive checking of the best models for both population size change and population divergence analyses showed good fits of the estimated models to the observed data (Figs S7, S8).

### Ecological niche modeling

**Fig. 6:**
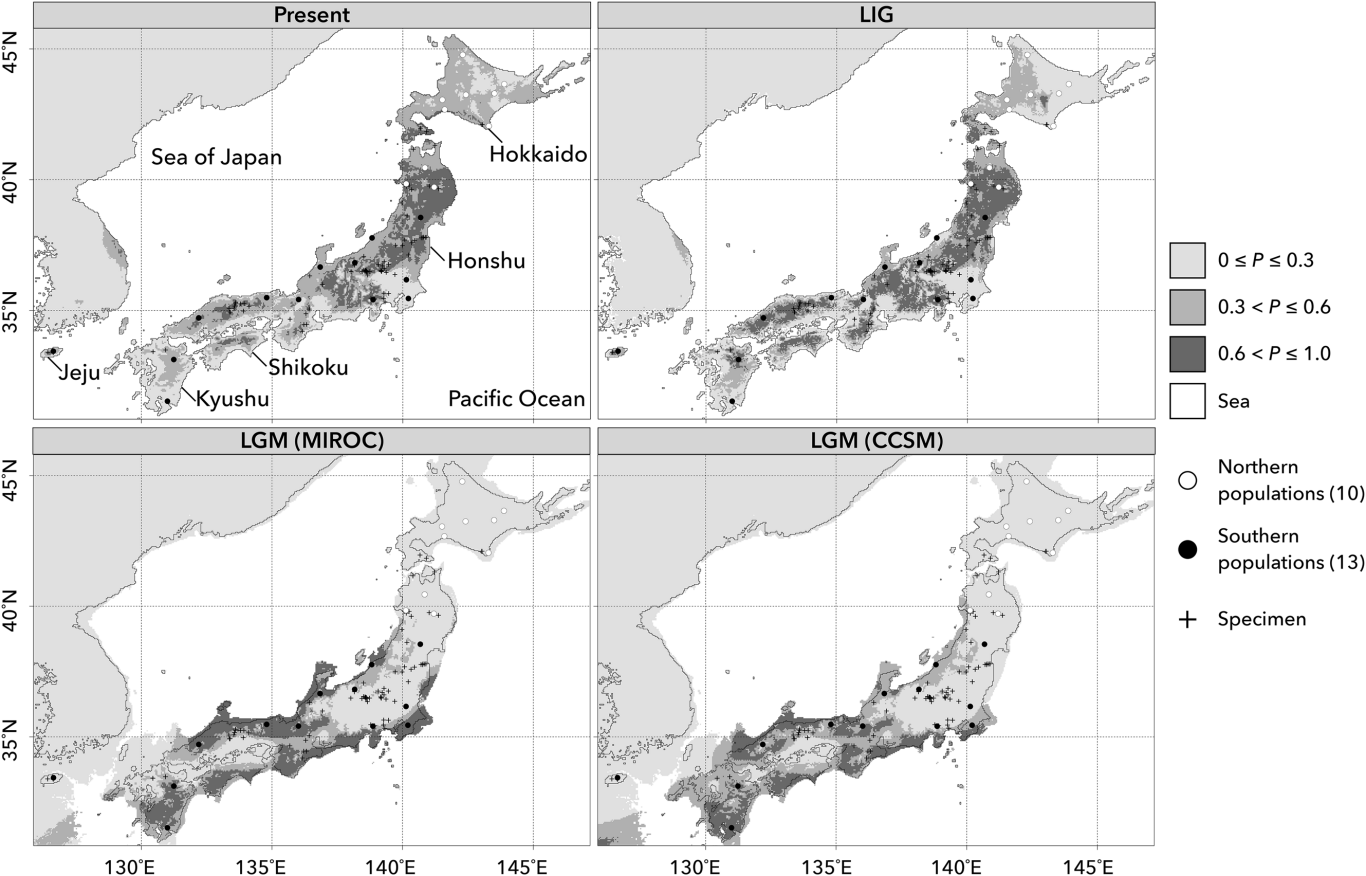
Inferred potential areas for *Magnolia kobus* at the present, the last inter-glacial (LIG, 130 kya) and the last glacial maximum (LGM, 21 kya) based on the community climate system model (CCSM) and the model for interdisciplinary research on climate (MIROC). *P* indicates probability of occurrence

The accuracy of ecological niche modeling was high with the average ± standard deviation of area under the curve (AUC) being 0.987 ± 0.002. The predicted distributions projected onto the current climate were a good fit to the species’ range except for Shikoku Island, where the species was predicted to be present currently as well as in LGM and LIG, but has no current populations of *M. kobus* (Figs. 1, 6). The climate variable that made the greatest contribution to the total variance was the precipitation in the coldest quarter (bio19, 58.1%). Using this model, potential distribution maps for the LGM (MIROC and CCSM) and LIG were created (Fig. 6).

Although under both LGM climate scenarios, several potential distribution areas in coastal regions south of 36°N for both Sea of Japan and Pacific Ocean sides were predicted with high probability (*P* > 0.6), the distribution areas with high probability north of 36°N were much smaller in CCSM than in MIROC. In Jeju, Honshu north of 41°N and Hokkaido, no potential distribution areas with *P* > 0.3 were detected. The predicted potential distribution areas in LIG and present were very similar.

## Discussion

### What factors contributed to producing the current genetic variation in *Magnolia kobus*?

*Magnolia kobus* showed a hierarchical genetic structure. At the highest level, two lineages, the northern and southern lineages, were identified and their geographical boundary was estimated to be at 39°N. The northern lineage consisted of two genetic clusters, whereas the southern one contained multiple genetic clusters. The level of genetic variation in nSSRs was significantly lower in the northern lineage than in the southern one. The cpDNA haplotype of the northern lineage was also fixed; there was only one haplotype, H. The *F*_ST_ value between the northern lineage and the common ancestral population at *K* = 2 was more than 100 times larger than that between the southern one and the ancestral population. Ecological niche modeling predicted that the probabilities of occurrence for this species in the northern area of Honshu (> 39°N) and in Hokkaido during the LGM were very low. These results suggest that genetic variation in the northern lineage has been affected by severe genetic drift during one or more past glacial periods. The significant genetic differentiation between the northern and southern lineages, and the lower level of genetic diversity in the northern lineage, that we observed in *M. kobus* are common trends among temperate tree species that are widely distributed along the Japanese archipelago (Hiraoka and Tomaru 2009; Sakaguchi et al. 2011; San Jose-Maldia et al. 2017; Tamaki et al. 2018; Tsumura et al. 2007, 2014). However, the locations of boundaries between northern and southern lineages are different among species. For example, the lineages of *Fagus crenata* and *Cryptomeria japonica* diverged between Sea of Japan and Pacific Ocean sides (Hiraoka and Tomaru 2009; Tsumura et al. 2014), those of *Kalopanax septemlobus* diverged gradually from central Honshu to Hokkaido (Sakaguchi et al. 2011), and those of *M. salicifolia* and *Quercus aliena* clearly diverged in the central Honshu (San Jose-Maldia et al. 2017; Tamaki et al. 2018). The boundary of two lineages of *M. kobus* is located in the northern part of Honshu and is rather geographically close to that of two lineages within *Pinus pumila* (Tani et al. 1996) and within *Betula maximowicziana* (Tsuda and Ide 2005, 2010), which are cold tolerant tree species and distributed further north.

The possibility of the existence of multiple past refugia in the southern part of Japan is supported by high genetic diversity in nSSRs, the existence of multiple genetic clusters in the southern lineage, endemic cpDNA haplotypes (haplotypes I and J) and several areas with high probabilities of potential distribution during the LGM (*P* > 0.6) on both Sea of Japan and Pacific Ocean sides in southern Japan. Although most of the populations of the southern lineage showed high genetic diversity and low *F*_ST_ values between each cluster and the ancestral population, two populations (20 and 23) did not. In the case of population 23 (Jeju), this population might have been affected by a severe historical isolation effect, as is suggested by the low probability of occurrence and the discontinuity with surrounding populations during both glacial and inter-glacial periods that were predicted by ecological niche modeling. Populations of *Rhododendron weyrichii*, which is a shrub growing in the warm temperate zone from Jeju to southern parts of Honshu Islands, on Jeju Island also showed long-term low effective population size and severe isolation as predicted by ABC and ecological niche modeling, respectively (Yoichi et al. 2016). Like *R. weyrichii*, *M. kobus* may have survived through a glacial period only in very restricted areas within Jeju Island.

From the comparison of three population size change models, histories of exponential growth from a small number of founders and stable population size were estimated for the northern and the southern lineage, respectively. These population size change histories selected by ABC are closely congruent with the hypotheses discussed in the paragraphs above based on diversity indices and predictions from ecological niche modeling. The posterior mode (95% HPD) of divergence time estimated by the isolation model was 11,300 (4,700–32,000) generations ago. To convert the divergence time into years ago, we must assume a generation time for *M. kobus*. It is reported that *M. kobus* starts flowering after 10–30 years from the seedling stage under garden conditions (Callaway 1994). As far as we know, there is no information about it in the wild. However, Takahashi et al. (2006) reported that average ± SD values for initial flowering and fruiting ages in the tree type of *Magnolia salicifolia*, which is a species related to *M. kobus*, were 17.6 ± 6.57 and 20.4 ± 6.70, respectively. Moreover, these authors reported that its maximum lifespan was more than 100 years and most individuals whose age was more than 50 years were well-grown trees at the canopy layer in the populations they studied. *M. kobus* can generally grow larger than *M. salicifolia*. With these factors taken into account, the generation time of *M. kobus* is considered to be 50 years or more. If we assume the generation time to be 50 years, the divergence time can be estimated at 565,000 (235,000–1,600,000) years ago. As we have made two major assumptions, of mutation rate and generation time, when estimating the divergence time, and since the estimated value has some degree of uncertainty denoted by the HPD, we should be cautious about its interpretation (Tsuda et al. 2016); however, the outcome suggests that the two lineages of *M. kobus* may have experienced several glacial-interglacial cycles after their divergence. *M. salicifolia*, which is also widely distributed in the Japanese archipelago, showed a population demographic history similar to that of the northern and the southern lineages, and a more distant divergence time than that in this study, but the extent of migrations between lineages after divergence was significant (Tamaki et al. 2018). These findings indicate that the current genetic diversities of temperate tree species that are broadly distributed in the Japanese archipelago may have been created not only by the effect of the most recent glacial period but also by the cumulative effects of several previous glacial periods. Model choice in population divergence analysis did not show significant migration between the two lineages after divergence. It can therefore be inferred that the refugia of the two lineages were different. Ecological niche modeling predicted a potential distribution area (0.3 < *P* ≤ 0.6) in LGM near the coast from 39° to 40°N on the Sea of Japan side, where the tail of the current distribution of the northern lineage is located, and it is considered to have been the glacial refugium for the northern lineage (Fig. 6). The possibility of a refugium near the coast of northern Japan on the Sea of Japan side was also reported for *C. japonica* (Kimura et al. 2014). In our study, populations near the boundary (10 and 11) showed more than 5% genetic admixture of the recessive cluster. This may be evidence of recent hybridization between the two lineages due to secondary contact. Similar admixture patterns have been also reported in *B. maximowicziana* almost at the same region (Tsuda et al. 2015). Moreover, the cited authors have indicated that the observed admixture pattern in *B. maximowicziana* was created not by secondary contact but by simple population split (Tsuda et al. 2015). To clarify whether the observed admixture pattern in *M. kobus* was created by secondary contact or simple population split, more intensive population sampling near the boundary would be needed.

### Relationship between geographical patterns of genetic variation and of leaf morphological traits

Populations from central Honshu on the Sea of Japan side to Hokkaido (1–10, 12–14 and 18) had wide leaves and large area. Populations with these leaf morphological traits corresponded well to *M. kobus* var. *borealis* (Ohashi 2015). Although the changes in leaf morphological traits were to some extent continuous and we could not identify varieties for some southern populations (Fig. 4), it was possible to confirm the existence of *M. kobus* var. *borealis*, which were distributed from the Sea of Japan side of central to northern Honshu and Hokkaido, based on these morphological characters. However, the genetic delimitation between lineages occurred at 39°N, further north than the morphological one (36°N). Because variety *borealis* recognized based on morphological characters comprises not only the northern lineage but also the southern one, we can conclude that it is not supported genetically and systematically.

Variety *borealis* appeared across both the northern and southern lineages. However, ABC analysis detected no significant historical migration between lineages after divergence. Population admixture was only detected in populations 11 and 17 for the southern lineage, but these populations showed leaf morphology of variety *kobus*. Therefore, it is unlikely that introgression has contributed to shape the morphology of variety *borealis* in some southern populations. We detected significant effects of environments on relative leaf width (PC1), the position of the maximum leaf width (PC2) and leaf area. Moreover, we also detected significant effects of population history on relative leaf width and leaf area. These results suggest that variation in the position of the maximum leaf width has been shaped by only natural selection, while variations in relative leaf width and leaf area have been shaped by both natural selection and population demographic history. However, it is possible that variations in these leaf morphological traits affected by natural selection are expressed through phenotypic plasticity and functional gene products (Ramírez-Valiente et al. 2010). In order to distinguish them a provenance test will be required.

### Conservation implications

Conservation units should be determined for *M. kobus* because there is clear genetic divergence between the northern and the southern lineages at ca. 39°N. Since the northern lineage consisted of single/two genetic clusters in nSSR (at *K* ≤ 6/*K* > 6, respectively), only one cpDNA haplotype and single variety, and the level of isolation by distance was relatively low, seed and/or seedling transfer may be permitted within the area north of 39°N, although the information about genetic differentiation of adaptive traits would be ideally needed to determine the range of seed and/or seedling transfer. On the other hand, since the southern lineage contained multiple genetic clusters, most populations showed clear genetic structure dominated by a single distinct genetic cluster in nSSR, composition of cpDNA haplotypes were different among populations and there were multiple varieties, it is obvious that seed/seedling transfer should be restricted even within the southern lineage and the optimal seed source would be the nearest natural population. In the southern lineage (< 39°N), for example, prefectural level conservation units may be practicable (prefectural borders are shown by dotted lines in Fig. 1). Many studies have reported the existence in natural forests of *M. kobus* seedlings that have escaped from trees planted near the forest (Fujii 1997; Ishida et al. 2008; Tamaki et al. 2016). To prevent genetic disturbance and to conserve genetic resources in natural forests, the provenance of seeds/seedlings should be considered carefully even when planting roadside trees especially in the southern distribution area.

## Electronic supplementary material

Below is the link to the electronic supplementary material.

Supplementary material 1 (PDF 787 kb)
