# Correction to: Population genetic structure and demography of *Magnolia kobus*: variety *borealis* is not supported genetically

**DOI:** 10.1007/s10265-020-01191-2

**Published:** 2020-04-29

**Authors:** Ichiro Tamaki, Naomichi Kawashima, Suzuki Setsuko, Jung-Hyun Lee, Akemi Itaya, Kyohei Yukitoshi, Nobuhiro Tomaru

**Affiliations:** 1 Gifu Academy of Forest Science and Culture, 88 Sodai, Mino, Gifu 501-3714 Japan; 2 Graduate School of Bioagricultural Sciences, Nagoya University, Furo-cho, Chikusa-ku, Nagoya, 464-8601 Japan; 3 Department of Forest Molecular Genetics and Biotechnology, Forestry and Forest Products Research Institute, Forest Research and Management Organization, 1 Matsunosato, Tsukuba, Ibaraki 305-8687 Japan; 4 Department of Biology Education, Chonnam National University, 77 Yongbong-ro, Buk-gu, Gwangju, 500-757 Republic of Korea; 5 Graduate School of Bioresources, Mie University, 1577 Kurimamachiya, Tsu, Mie 514-8507 Japan; 6 Present Address: Mie Prefecture Forestry Research Institute, 3769-1 Nihongi, Hakusan-cho, Tsu, Mie 515-2602 Japan

## Correction to: Journal of Plant Research (2019) 132:741–758 https://doi.org/10.1007/s10265-019-01134-6

The article Population genetic structure and demography of *Magnolia kobus*: variety *borealis* is not supported genetically, written by Kohma Ichiro Tamaki, Naomichi Kawashima, Suzuki Setsuko, Jung Hyun Lee, Akemi Itaya, Kyohei Yukitoshi and Nobuhiro Tomaru, was originally published Online First without Open Access. After publication in volume 132, issue 6, page 741–758 the author decided to opt for Open Choice and to make the article an Open Access publication. Therefore, the copyright of the article has been changed to © The Author(s) 2020 and the article is forthwith distributed under the terms of the Creative Commons Attribution 4.0 International License (https://creativecommons.org/licenses/by/4.0/), which permits use, duplication, adaptation, distribution and reproduction in any medium or format, as long as you give appropriate credit to the original author(s) and the source, provide a link to the Creative Commons license, and indicate if changes were made.

The original article was updated.

