# Supplementary material for: Population genetic structure and demography of Magnolia kobus: variety borealis is not supported genetically
Source: J Plant Res. 2019 Sep 5;132(6):741–58. doi: 10.1007/s10265-019-01134-6 (PMC7196954; doi:10.1007/s10265-019-01134-6)
Supplement: Supplementary file 1 — Supplementary material 1 (PDF 787 kb) [file 10265_2019_1134_MOESM1_ESM.pdf]

Electronic supplementary materials

Title:

Population genetic structure and demography of *Magnolia kobus*: variety *borealis* is not supported genetically

Authors:

Ichiro Tamaki, Naomichi Kawashima, Kyohei Yukitoshi, Jung-Hyun Lee, Suzuki Setsuko, Akemi Itaya, and Nobuhiro Tomaru

Journal:

Journal of Plant Research

Corresponding author:

Nobuhiro Tomaru
Graduate School of Bioagricultural Sciences, Nagoya University, Furo-cho, Chikusa-ku, Nagoya 464-8601, Japan
Tel: +81-52-789-4048
Fax: +81-52-789-5014
E-mail: tomaru@agr.nagoya-u.ac.jp

Contents:

Tables S1–S4
Figs. S1–S8

Table S1 Prior distributions of parameters for population size change and population divergence analyses

Analysis	Parameter	Distribution
Population size change	N_{CUR}	Uniform (10^3, 1.5×10^5)
	G	Uniform (-0.001, 0)
	T	Uniform (1, 5×10^4)
	RN_{ANC} [a]	Uniform (1, 20)
Population divergence	N_{N}	Uniform (10^3, 1.5×10^5)
	N_{S}	Uniform (10^3, 1.5×10^5)
	G	Fixed to -2.04×10^{-4}
	T_{DIV}	Uniform (1, 5×10^4)
	Nm_{NS}	Uniform (1, 20)
	Nm_{SN}	Uniform (1, 20)
	β	Uniform (0, 1)
Common	mean μ	Log-uniform (10^{-5}, 10^{-3})
	shape	Uniform (0.5, 5)
	mean P_{GSM}	Uniform (0, 1)

[a] $N_{\mathrm{ANC}} = N_{\mathrm{CUR}} \times RN_{\mathrm{ANC}}$

Table S2 Nucleotide sequence variation among three haplotypes (H to J) from *Magnolia kobus* and eight outgroup haplotypes consisting of seven haplotypes from *M. salicifolia* (A to G) and one haplotype from *M. denudata* in four chloroplast DNA regions

			*trn*S–*trn*G (684 bp)					*trn*T–*psb*D (1461 bp)										
Species	Haplotype	*N*	114	358	452	546	552	730	941	1026	1194	1232	1805	1825	1999	2041	2051	2089
M. salicifolia	A		-	T	G	G	T	A	G	G	T	C	T	G	G	G	A	A
M. salicifolia	B		-	•	•	•	•	•	•	•	•	•	•	A	•	•	•	•
M. salicifolia	C		-	•	•	•	•	•	•	•	•	•	•	A	•	•	•	•
M. salicifolia	D		-	•	•	•	•	•	•	•	•	•	•	•	•	•	•	•
M. salicifolia	E		-	•	•	•	•	•	•	•	C	•	•	•	•	•	•	•
M. salicifolia	F		-	•	•	•	•	•	•	T	•	•	•	•	•	•	•	G
M. salicifolia	G		I_1	•	•	•	•	•	•	T	•	•	•	•	•	A	•	G
M. kobus	H	35	-	•	•	T	G	G	•	T	•	•	•	•	•	•	•	G
M. kobus	I	11	-	•	•	T	G	•	•	T	•	•	•	•	•	•	•	G
M. kobus	J	2	-	C	•	T	G	•	•	T	•	T	•	•	•	•	•	G
M. denudata			-	•	A	•	•	•	A	T	•	•	C	•	A	•	G	G

Table S2 continued

			*trn*T–*trn*L (681 bp)														*rpl*36–*inf*A–*rps*8–*rpl*14 (1106 bp)			
Species	Haplotype	*N*	2161	2172	2173	2219	2263	2265	2296	2300	2357	2408	2636	2653	2736	2743	3249	3276	3449	3854
M. salicifolia	A		A	A	A	A	A	I_2	G	G	G	G	T	G	A	A	A	G	G	•
M. salicifolia	B		•	•	•	•	•	I_2	•	•	•	•	•	•	•	•	•	•	•	•
M. salicifolia	C		•	•	C	•	•	I_2	•	•	•	•	•	•	•	•	•	•	•	•
M. salicifolia	D		•	C	•	•	•	I_2	•	•	•	•	•	•	•	•	•	•	•	•
M. salicifolia	E		•	•	•	•	•	I_2	•	•	•	•	•	•	•	•	•	•	•	•
M. salicifolia	F		•	•	•	•	•	I_2	•	•	•	•	•	•	•	•	•	A	•	•
M. salicifolia	G		C	•	•	•	•	I_2	•	•	T	•	•	•	•	G	•	•	•	•
M. kobus	H	35	•	•	•	•	G	I_2	•	•	T	•	•	•	G	•	•	•	A	•
M. kobus	I	11	•	•	•	G	G	I_2	•	•	T	•	•	•	G	•	•	•	A	•
M. kobus	J	2	•	•	•	G	G	I_2	•	•	T	•	•	•	G	•	•	•	A	•
M. denudata			•	•	•	•	•	-	T	T	T	T	C	T	•	•	G	•	•	I_3

N, number of individuals; •, the same base as in haplotype A; -, deletion; I_1, insertion of TTATCTTTCTTTTCTTTATTCTAT; I_2, insertion of CTATAA; I_3, insertion of GAGAA. Sequence data for *M. salicifolia* and *M. denudata* are the same as those used in Tamaki et al. (2018). Gray columns indicate sites variable within *M. kobus*.

Table S3 Principal components (PCs) of leaf shape estimated by SHAPE

Principal component	Eigenvalue ($\times 10^{-3}$)	Contribution (%)	Cumulative contribution (%)
PC1	3.93	56.16	56.16
PC2	1.11	15.86	72.02
PC3	0.83	11.79	83.81
Overall	7.00		

Only the three PCs whose contribution to the overall variance was more than 5% are shown.

Table S4 Proportion of models correctly predicted and classification error rate estimated by random forest (RF)

		Simulated	Predicted model							Classification
Analysis	Lineage	model	SNM	PGM	SRM	ISM	IMM	IMM_{NS}	IMM_{SN}	error rate
Population size change	Northern	SNM	**0.790**	0.108	0.103	–	–	–	–	0.210
		PGM	0.104	**0.891**	0.004	–	–	–	–	0.109
		SRM	0.234	0.026	**0.740**	–	–	–	–	0.260
	Southern	SNM	**0.801**	0.104	0.096	–	–	–	–	0.199
		PGM	0.111	**0.883**	0.006	–	–	–	–	0.110
		SRM	0.232	0.028	**0.740**	–	–	–	–	0.260
Population divergence		ISM	–	–	–	**0.790**	0.037	0.144	0.029	0.210
		IMM	–	–	–	0.005	**0.664**	0.181	0.149	0.336
		IMM_{NS}	–	–	–	0.068	0.227	**0.666**	0.039	0.334
		IMM_{SN}	–	–	–	0.048	0.262	0.104	**0.587**	0.413

Predictions by RF were composed of 1,000 trees based on a trained set of 10,000 simulated predictor variables (summary statistics). The response variable of RF was the demographic model. Proportions of correctly predicted demographic models are in bold face.

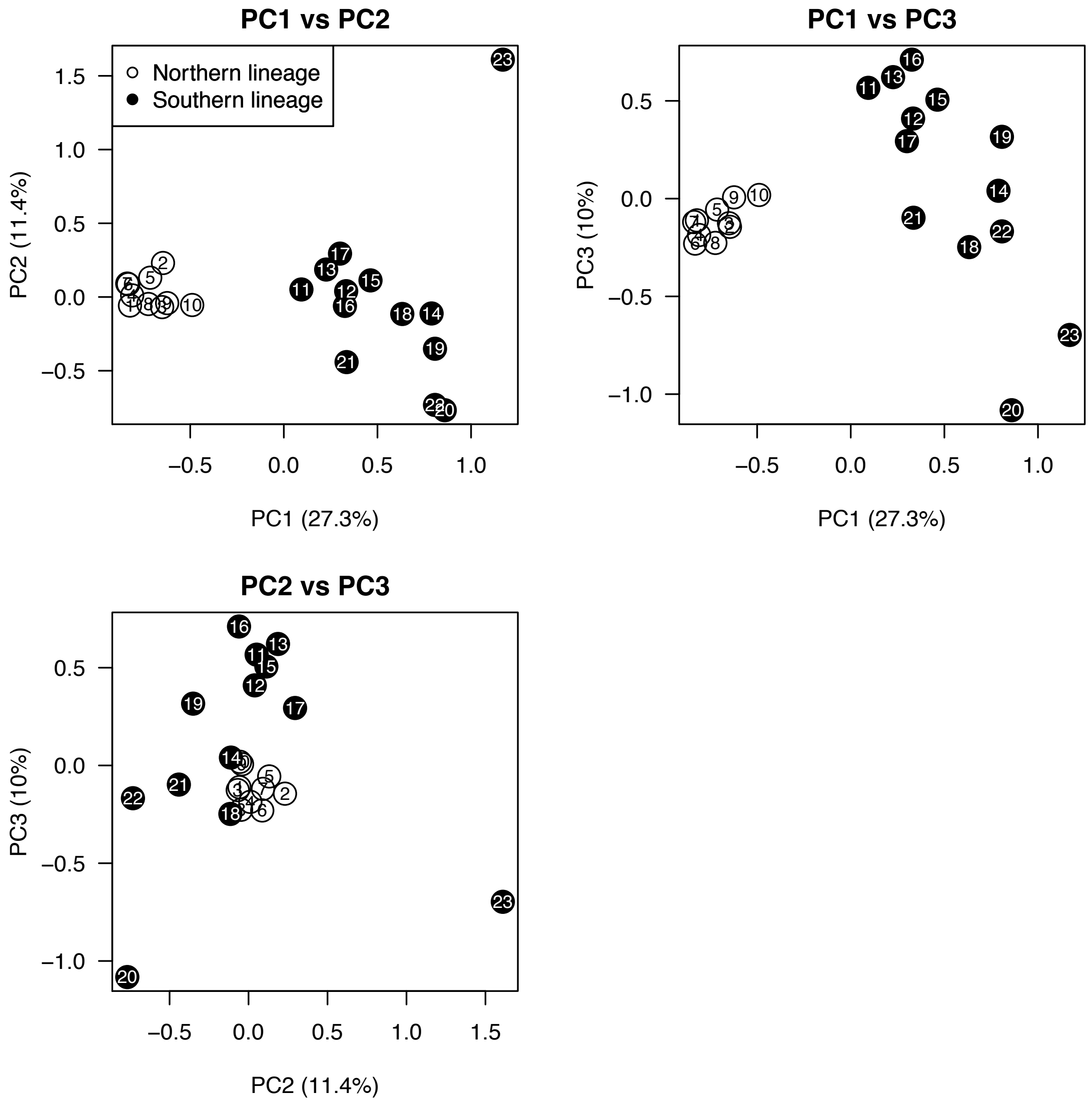

Fig. S1 Distributions of principal components estimated by allele frequencies for 23 *Magnolia kobus* populations. Numbers indicate populations listed in Table 1.

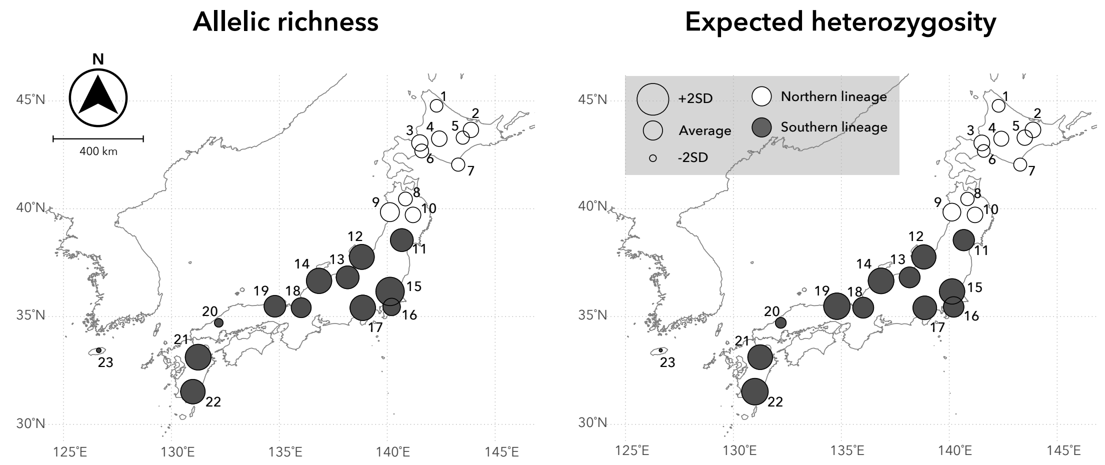

Fig. S2 Distributions of allelic richness and expected heterozygosity of 23 *Magnolia kobus* populations. Numbers indicate populations listed in Table 1.

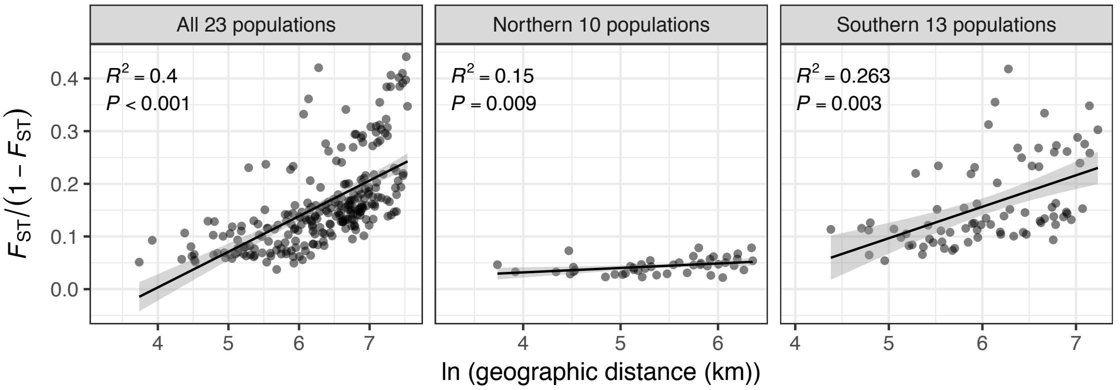

Fig. S3 Relationships between geographical and genetic distances for 23 *Magnolia kobus* populations.

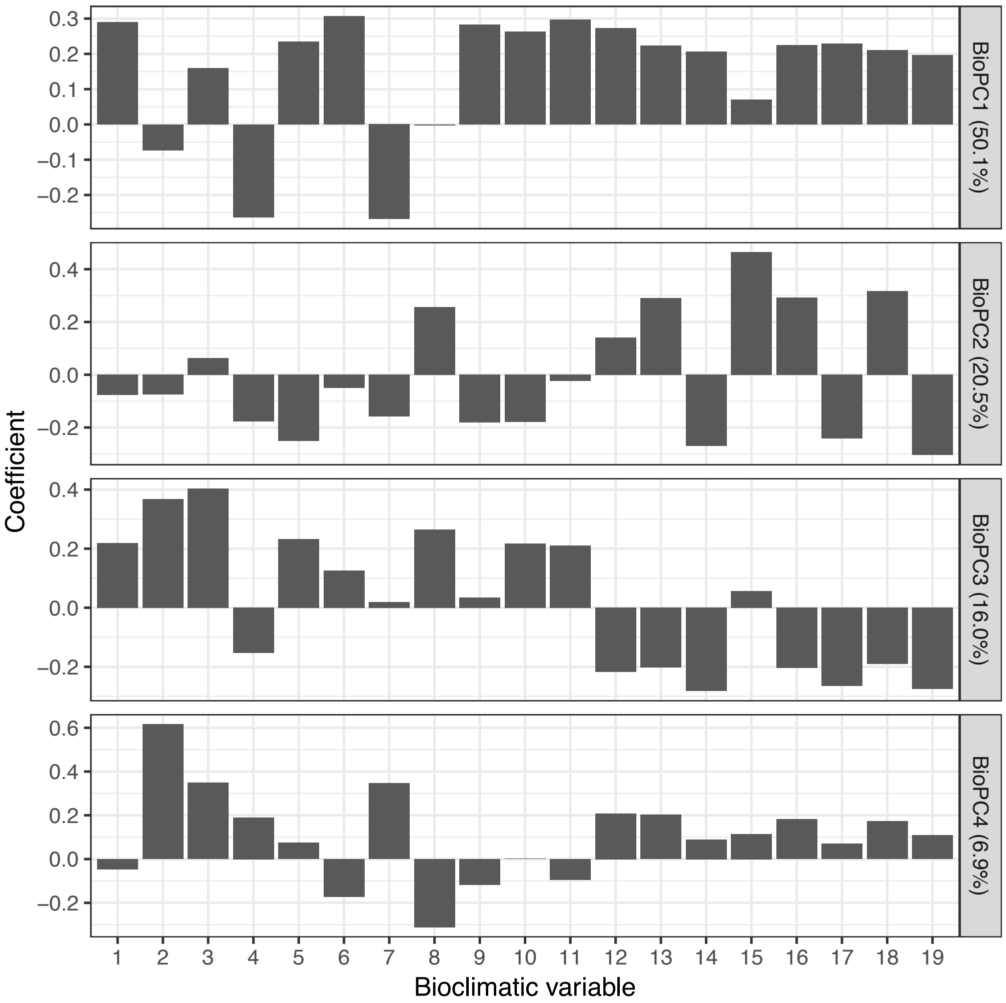

Fig. S4 Coefficients of bioclimatic variables for each principle component (BioPC). BioPCs whose contribution was more than 5% are shown. bio1, annual mean temperature; bio2, mean diurnal range [mean of monthly (max temp - min temp)]; bio3, isothermality (bio2 / bio7 × 100); bio4, temperature seasonality (standard deviation × 100); bio5, max temperature of warmest month; bio6, min temperature of coldest month; bio7, temperature annual range (bio5 - bio6); bio8, mean temperature of wettest quarter; bio9, mean temperature of driest quarter; bio10, mean temperature of warmest quarter; bio11, mean temperature of coldest quarter; bio12, annual precipitation; bio13, precipitation of wettest month; bio14, precipitation of driest month; bio15, precipitation Seasonality (coefficient of variation); bio16, precipitation of wettest quarter; bio17, precipitation of driest quarter; bio18, precipitation of warmest quarter; bio19, precipitation of coldest quarter.

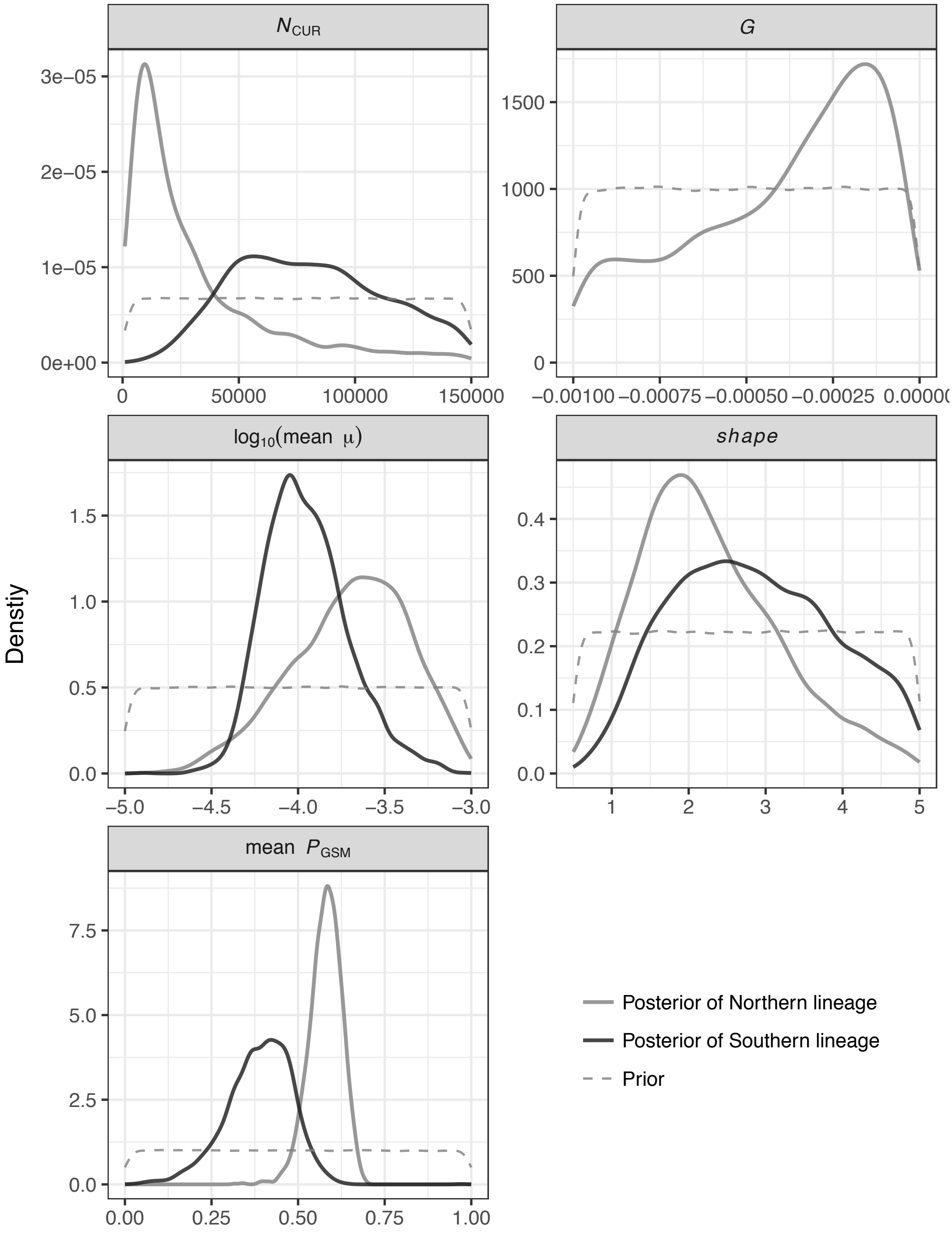

Fig. S5 Posterior and prior distributions of parameters in population size change analysis. N_{CUR}, current effective population size; *G*, population growth rate; mean *μ*, *shape* and mean P_{GSM}, parameters in generalized stepwise mutation model for nuclear microsatellites. Unit of effective population size is the number of diploid individuals.

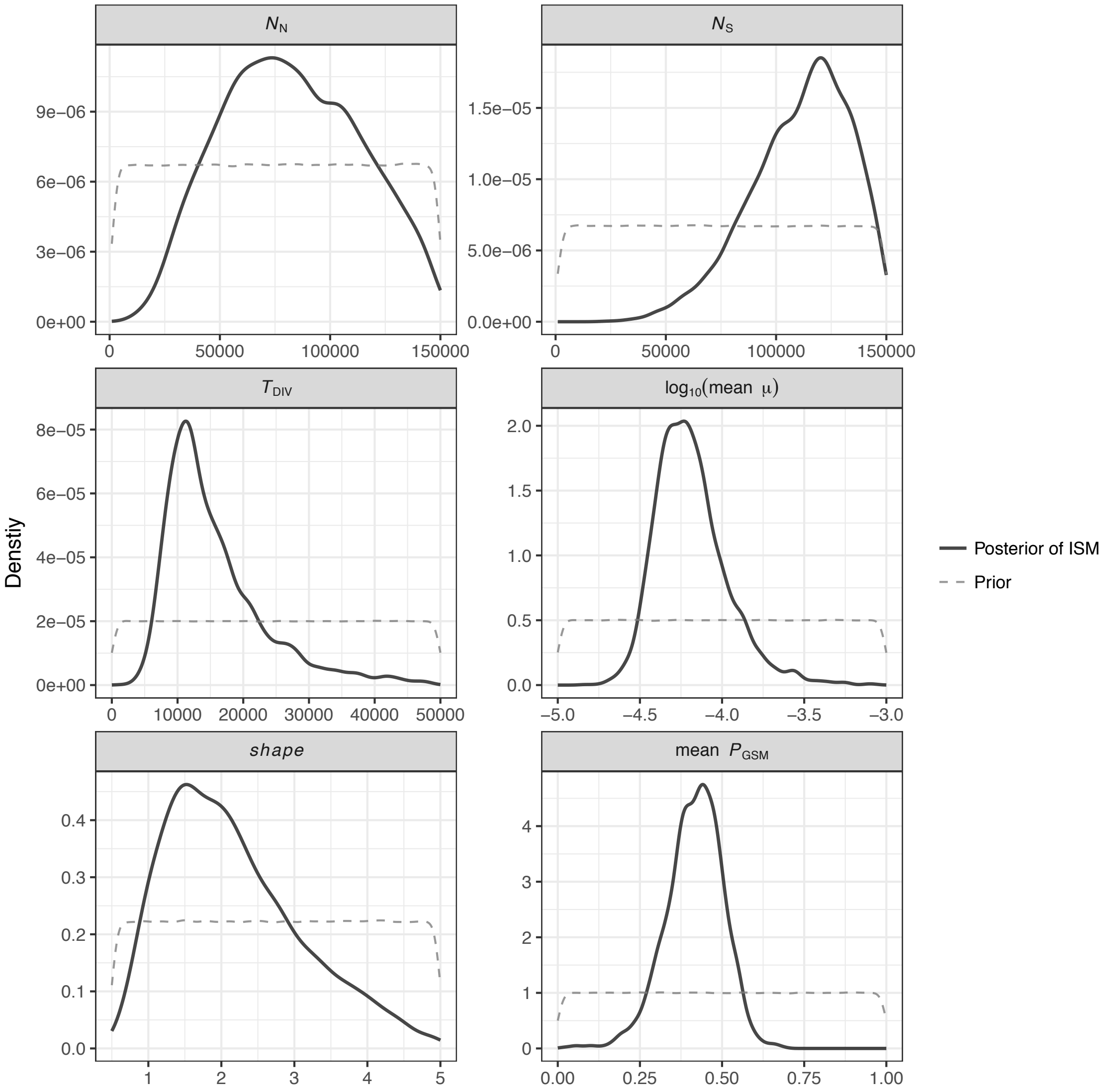

Fig. S6 Posterior and prior distributions of parameters in the best model, the isolation model. N_{N} and N_{S}, current effective population sizes of the northern and the southern lineages, respectively; T_{DIV}, divergence time; mean μ, *shape* and mean P_{GSM}, parameters in the generalized stepwise mutation model for nuclear microsatellites. Units of effective population size and divergence time are the numbers of diploid individuals and generations, respectively.

(A)

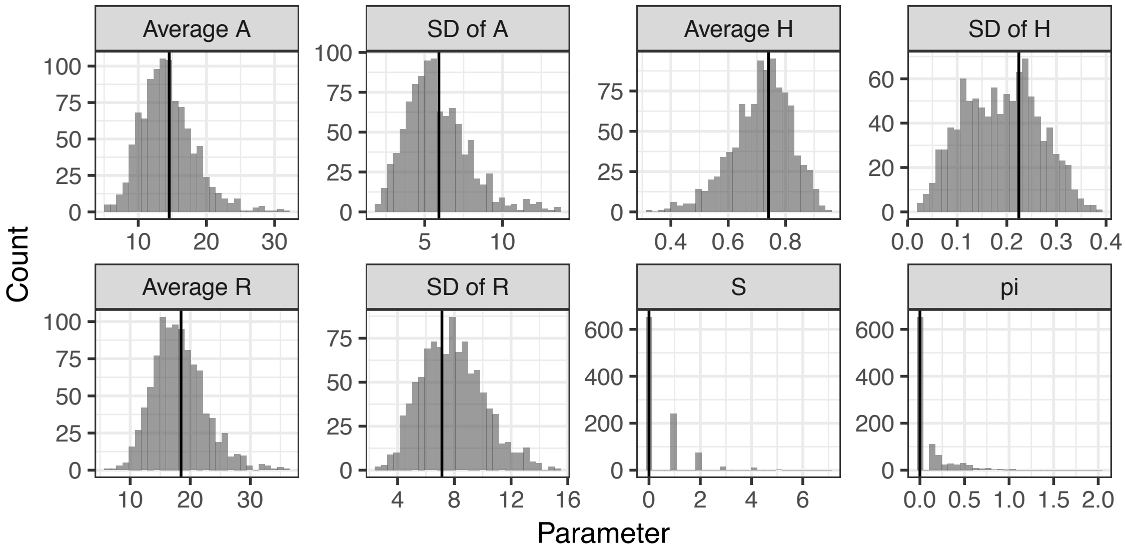

(B)

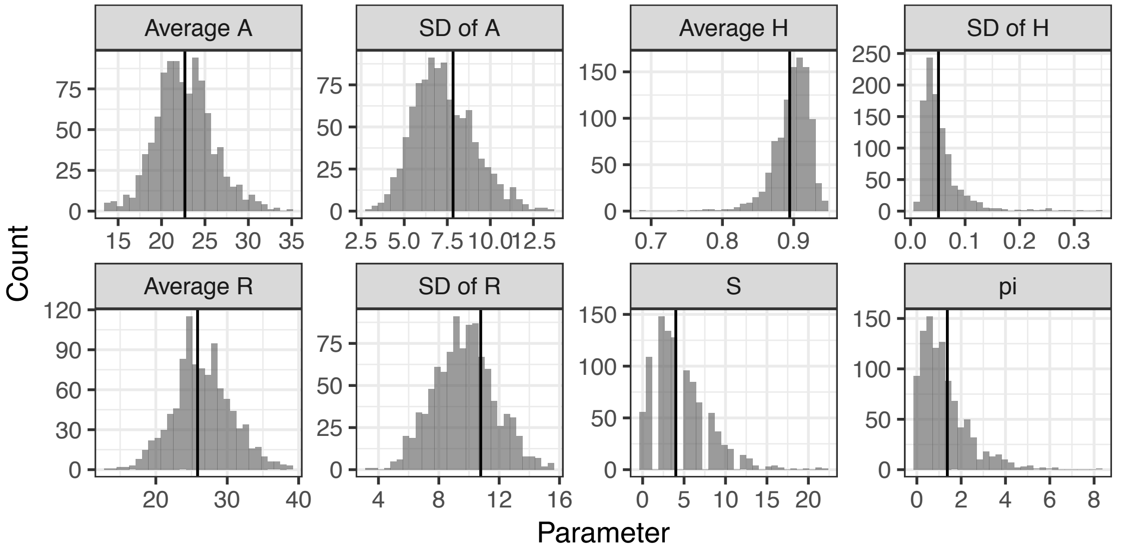

Fig. S7 Predicted and observed values (histogram and vertical bar, respectively) for the northern and the southern lineages in population size change analysis (A and B, respectively). Posterior predictive simulation was performed using the best model. *A*, number of alleles; *H*, expected heterozygosity; *R*, allele size range; *S*, number of polymorphic sites; *pi*, mean number of pairwise differences.

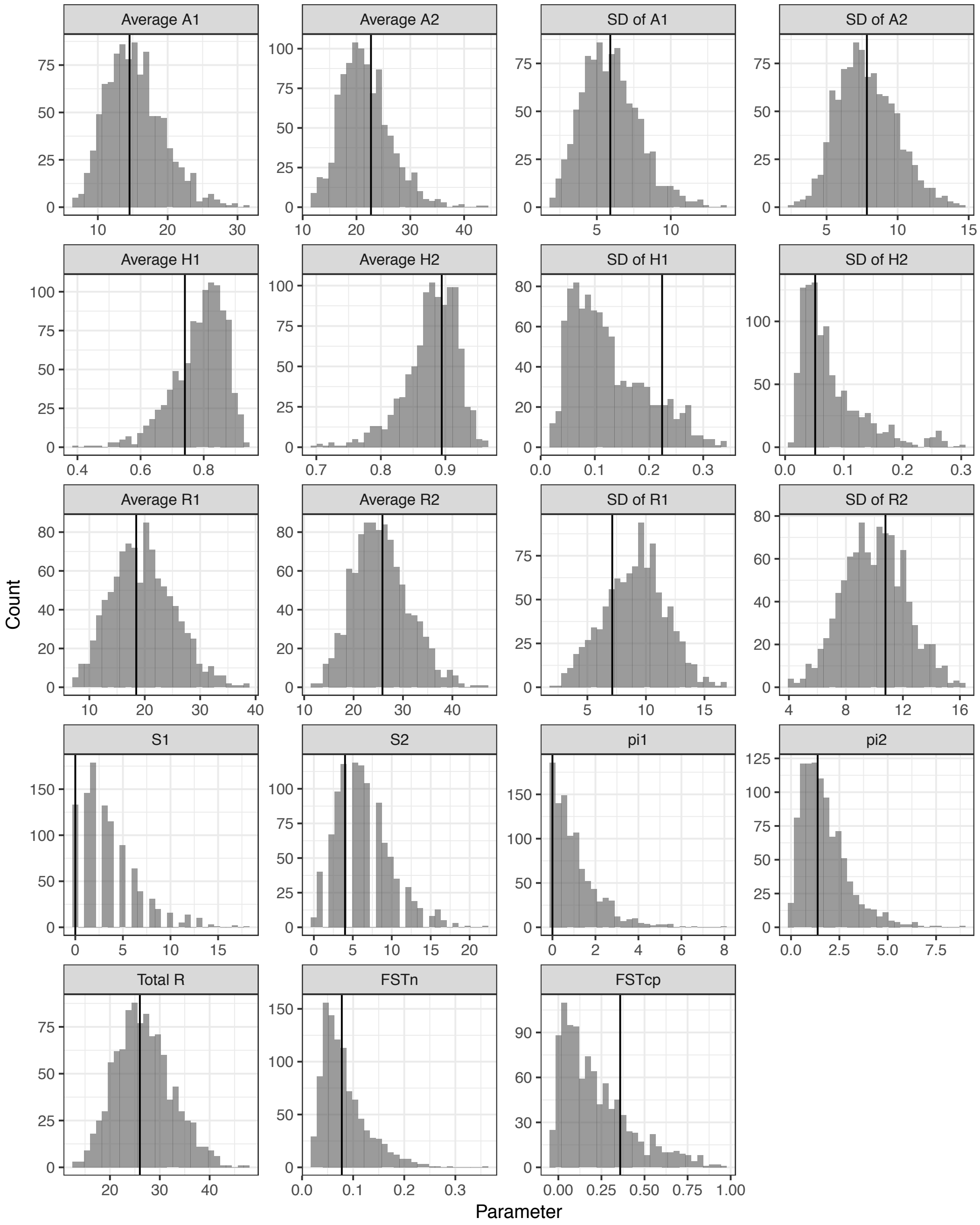

Fig. S8 Predicted and observed values (histogram and vertical bar, respectively) in population divergence analysis. Posterior predictive simulation was performed using the best model, the isolation model. *A*, number of alleles; *H*, expected heterozygosity; *R*, allele size range; *S*, number of polymorphic sites; *pi*, mean number of pairwise differences; Total R, allele size range for samples overall; *FSTn*, F_{ST} for over all loci of 13 nuclear microsatellites; *FSTcp*, F_{ST} for chloroplast DNA haplotypes. Diagrams for the northern and the southern lineages are indicated by 1 and 2, respectively.
